# Antioxidant and anticancer properties of citrus-mediated nanoformulations revealed by meta-analysis

**DOI:** 10.1038/s41598-025-15291-3

**Published:** 2025-09-26

**Authors:** Rahmat Budiarto, Mohammad Miftakhus Sholikin, Danung Nur Adli, Teguh Wahyono, Temoor Ahmed, Tri Ujilestari, Hayssam M. Ali

**Affiliations:** 1 Department of Agronomy, Faculty of Agriculture, Universitas Padjadjaran, Sumedang, 45363 Indonesia; 2 Research Center for Animal Husbandry, National Research and Innovation Agency (BRIN), Bogor, 16915 Indonesia; 3 Smart Livestock Industry Study Programme, Department of Feed and Animal Nutrition, Faculty of Animal Science, Universitas Brawijaya, Malang, East Java 65145 Indonesia; 4 Research Center for Food Technology and Processing, National Research and Innovation Agency (BRIN), Gunungkidul, 55861 Indonesia; 5 Institute of Biotechnology, Zhejiang University, Hangzhou, 310058 China; 6 Department of Plant Biotechnology, Korea University, Seoul, 02481 South Korea; 7 Department of Botany and Microbiology, College of Science, King Saud University, Riyadh, 11421 Saudi Arabia

**Keywords:** Antioxidant activity, Cancer cell cytotoxicity, Citrus-mediated nanoformulations, Citrus peel extract, Silver nanoparticles, Cancer therapy, Plant sciences

## Abstract

This study aims to explore and analyse the potential antioxidant and anticancer potential of various citrus-mediated nanoformulations (CMNs), focusing on their effectiveness in scavenging free radicals and inducing cytotoxicity in cancer cells. This research employs a meta-analysis approach to assess data from multiple studies on CMNs. This study is the first meta-analysis to evaluate the antioxidant and anticancer properties of CMNs concurrently. This study offers a novel perspective by examining citrus species, plant parts utilised, nanoparticle types, particle sizes, and coating materials. The analysis employs the Population, Intervention, Comparison, and Outcome (PICO) framework and complies with the Preferred Reporting Items for Systematic Reviews and Meta-Analyses (PRISMA) guidelines. The analysis utilizes Hedges’ effect size and includes validation through fail-safe N. The IC_50_ evaluation (µg/mL) revealed a significant effect of CMNs on antioxidant activity (d_++_ = 3.49; *P* < 0.05). The IC50 value of 3.49 in the CMN indicates that a lower concentration is sufficient to inhibit 50% of the free radical activity, reflecting a stronger antioxidant potential than that of the control group. However, the overall antioxidant assay results (d_++_ = 0.2; *P* = 0.309) and radical inhibition (%) for CMNs (d_++_ = 0.1; *P* = 0.602) did not significantly differ. Subgroup analysis provided further insights, showing that both citrus peel and polyvinyl alcohol significantly reduced IC_50_ values (d_++_ >1; *P* < 0.05). In addition, radical inhibition significantly increased in CMNs derived from *Citrus paradisi* (d_++_ = 3.05; *P* = 0.015), followed by those derived from *Citrus limon* (d_++_ = 2.25; *P* < 0.01) and *Citrus reticulata* (d_++_ = 1.03; *P* = 0.025). Various types of nanoformulations, such as Ag chitosan-NP (silver nanoparticle with chitosan), Ag-NP (silver nanoparticles), cerium dioxide nanoparticle (CeO₂-NPs), hydrogel-based nanocomposite (Hydrogel-NPCs), pectin-based nanoemulsion (Pectin-NPEs), titanium dioxide nanoparticle (TiO₂-NP), and whey-based nanoemulsion (Whey-NPEs), also significantly enhanced free radical scavenging activity (d_++_ >1; *P* < 0.01). In terms of anticancer activity, CMN has a strong effect size (|d_++_| >1; *P* < 0.05), with species such as *Citrus macroptera* and plant parts such as juice showing highly positive effects (d_++_ = 2.25; *P* < 0.001). Additionally, nanoparticles with sizes between 101 and 500 nm exhibited significant effectiveness (d_++_ = 2.26; *P* < 0.001). These findings indicate that citrus-derived compounds have potential as anticancer agents by actively enhancing the antioxidant capacity of healthy cells. The significant antiproliferative activity observed across multiple cancer cell lines, supported by robust statistical analyses, demonstrates the potential of CMNs as a natural therapeutic approach for cancer prevention and treatment.

## Introduction

Growing health awareness has intensified the search for safer and more effective therapeutic and preventive alternatives. Natural compounds derived from plants, particularly those from the genus *Citrus*, have demonstrated significant potential in diverse health applications. Citrus fruits are rich in bioactive compounds, such as ascorbic acid, flavonoids, and phenolic acids, which are known for their antioxidant, antimicrobial, and anticancer properties^1–4^. However, the limited bioavailability and stability of these compounds often pose a challenge to their use as therapeutic agents. To address this limitation, current research focuses on nanotechnology-based approaches, particularly the development of nanoparticles synthesized from citrus extracts^5,6^. These nanoparticles are expected to enhance the effectiveness of bioactive compounds through improved formulation and increased absorption^7^.

Nanoparticles derived from natural sources, such as citrus fruits, present numerous advantages, including safety and sustainability. Research has revealed that biogenic nanoparticles have significant potential as carriers for bioactive compounds^8^increasing their delivery to target cells^9,10^. The antioxidant and anticancer activities of CMNs are expected to provide valuable insights for the development of more effective alternative therapies. Current studies investigating the antioxidant activity of biogenic nanoparticles have shown variable results, with some demonstrating significant antioxidant properties and others showing limited efficacy^11–13^. These findings suggest that CMNs have the potential to reduce oxidative stress, which often contributes to the development of degenerative diseases and cancer. Furthermore, incorporating CMNs into therapeutic applications could increase the bioavailability of essential nutrients and phytochemicals found in citrus fruits. By improving the efficacy of these compounds, CMNs may play a crucial role in preventive healthcare strategies. Continued research in this area could lead to innovative treatments that utilize the natural properties of CMNs for improved health outcomes.

Furthermore, the anticancer activity of CMNs is strong and significant particularly in reducing cancer cell viability^14–16^. These findings are particularly promising, especially considering the challenges associated with conventional cancer treatments, which often involve adverse side effects. Recent findings indicate that ascorbic acid and flavonoids from citrus can induce apoptosis and inhibit the proliferation of cancer cells^1,17^. In this context, the development of nanoparticles based on citrus extracts may increase the absorption of these compounds into cancer cells, thereby increasing therapeutic efficacy and minimizing side effects.

A meta-analysis of data on the antioxidant and anticancer activities of citrus plays a crucial role in systematically evaluating and quantifying the factors that affect its effectiveness^18,19^. These factors include the citrus species, the plant part used, the type and size of the nanoparticle formulation, the coating materials, and the specific cancer cell line or type. By synthesizing data from multiple studies, a meta-analysis provides more reliable and generalizable conclusions than those drawn from individual studies alone. Comparative analyses with positive controls such as Trolox demonstrated that nanoparticle type, size, and the chemical composition of citrus extracts are critical determinants of therapeutic efficacy^20,21^. These findings create opportunities for further research on optimizing CMNs, which could enhance the therapeutic effects of the bioactive compounds contained within. This study hypothesizes that nanoformulations derived from citrus phytochemicals, demonstrate stronger antioxidant and anticancer activities than both positive controls (butylated hydroxytoluene and doxorubicin) and negative controls (non-nano citrus extract and untreated cancer cells). Through systematic evaluation of multiple cancer cell lines and nanoparticle formulations, we investigated the dual efficacy of these nanoformulations in reducing oxidative stress and inhibiting cancer cell proliferation.

The primary objective of this study was to evaluate the effectiveness and mechanisms of action of CMNs in the context of health, as well as to provide data supporting the development of CMN-based therapies as safe and effective alternatives for cancer treatment. Therefore, this research aims to identify the potential of nanoparticles in combating degenerative diseases and cancer through antioxidant and anticancer mechanisms. This study has the potential to make significant contributions to public health and the development of improved natural health products.

## Materials and methods

### Topic selection, literature search, and literature evaluation

**Fig. 1:**
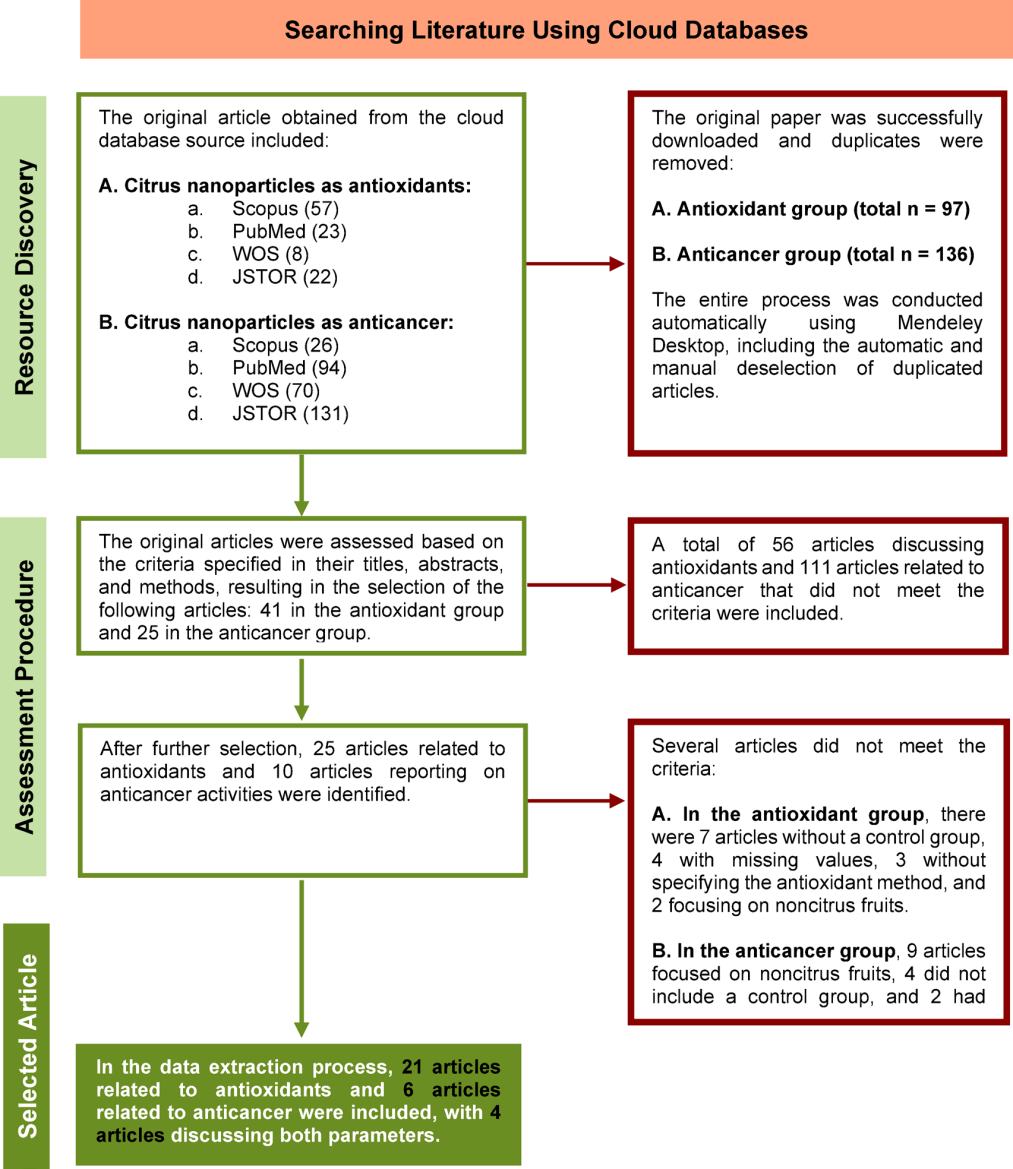
Process of discovering, assessing, and selecting original articles on the antioxidant and anticancer activities of nanoparticles from citrus phytochemicals.

The topic was selected on the basis of the framework of previous meta-analyses, utilizing the Population, Intervention, Comparison, and Outcome (PICO) system and following the Preferred Reporting Items for Systematic Reviews and Meta-Analyses (PRISMA) guidelines^22,23^. The topic description based on PICO was as follows: P = in vitro experiments on the measurement of antioxidant and anticancer activities, I = the level or dosage of CMN affecting the measurements, C = negative control (citrus extract or others) and positive control (standard compounds used as antioxidants and anticancer agents), and O = the resultant values of antioxidant and anticancer activity. The PICO framework was then used to generate search keywords for cloud databases, including Scopus, PubMed, WoS, and JSTOR. The detailed search results are presented in Table 1. The search yielded 110 articles related to antioxidants and 321 articles related to anticancer agents. Duplicate data were removed via Mendeley Desktop version 1.19.8, resulting in 97 articles related to antioxidants and 136 articles related to anticancer agents. Further selection was conducted using the following criteria: (a) original research articles published in English and indexed with a digital object identifier (DOI); (b) inclusion of treatment versus control groups; (c) use of nanoparticles derived from citrus fruits (with unspecified varieties); and (d) reported results on antioxidant and/or anticancer activities. The final selection included 21 articles on antioxidants, 6 articles on anticancer agents, and 4 articles covering both parameters. The article selection process is illustrated in Fig. 1.

**Table 1:** Original research investigations from cloud databases.

Year	CloudD	Keywords	Total	ORI	REV	Others
Antioxidant						
2011–2024	Scopus	TITLE-ABS-KEY (citrus* OR orange*) AND TITLE-ABS-KEY (nanoparticle* OR nanoformulation*) AND TITLE-ABS-KEY (antioxidant OR anti-oxidant*)	59	57	0	2
2014–2024	PubMed	ALL FIELDS (citrus* OR orange*) AND ALL FIELDS (nanoparticle* OR nanoformulation*) AND ALL FIELDS (antioxidant OR anti-oxidant*)	24	23	0	1
2018–2023	WoS	TOPIC (citrus* OR orange*) AND TOPIC (nanoparticle* OR nanoformulation*) AND TOPIC (antioxidant OR anti-oxidant*)	8	8	0	0
2001–2019	JSTOR	ALL FIELDS (citrus* OR orange*) AND ALL FIELDS (nanoparticle* OR nanoformulation*) AND ALL FIELDS (antioxidant OR anti-oxidant*)	22	22	0	0
Anticancer						
2015–2024	Scopus	TITLE-ABS-KEY (citrus* OR orange*) AND TITLE-ABS-KEY (nanoparticle* OR nanoformulation*) AND TITLE-ABS-KEY (anticancer* OR anti-cancer*)	29	26	2	1
2011–2024	PubMed	ALL FIELDS (citrus* OR orange*) AND ALL FIELDS (nanoparticle* OR nanoformulation*) AND ALL FIELDS (anticancer* OR anti-cancer*)	140	94	9	37
2008–2024	WoS	TOPIC (citrus* OR orange*) AND TOPIC (nanoparticle* OR nanoformulation*) AND TOPIC (anticancer* OR anti-cancer*)	72	70	2	0
2000–2021	JSTOR	ALL FIELDS (citrus* OR orange*) AND ALL FIELDS (nanoparticle* OR nanoformulation*) AND ALL FIELDS (anticancer* OR anti-cancer*)	170	131	1	38

The search matrix is applied to the title, abstract, and keywords (TITLE-ABS-KEY) of the articles, as well as the entire article (ALL FIELDS). The connectors “OR” and “AND” denote logical relationships, whereas the notation (*) represents all variations of the word that follows it.

CloudD is the cloud database source used; JSTOR, accessed at https://www.jstor.org/; ORI, original research article; PubMed, sourced from https://pubmed.ncbi.nlm.nih.gov/; REV, review articles or meta-analyses and similar types; Scopus, sourced from https://www.scopus.com/; WoS, sourced from https://www.webofscience.com/.

### Estimation of risk of bias (ROB) and data extraction

**Fig. 2:**
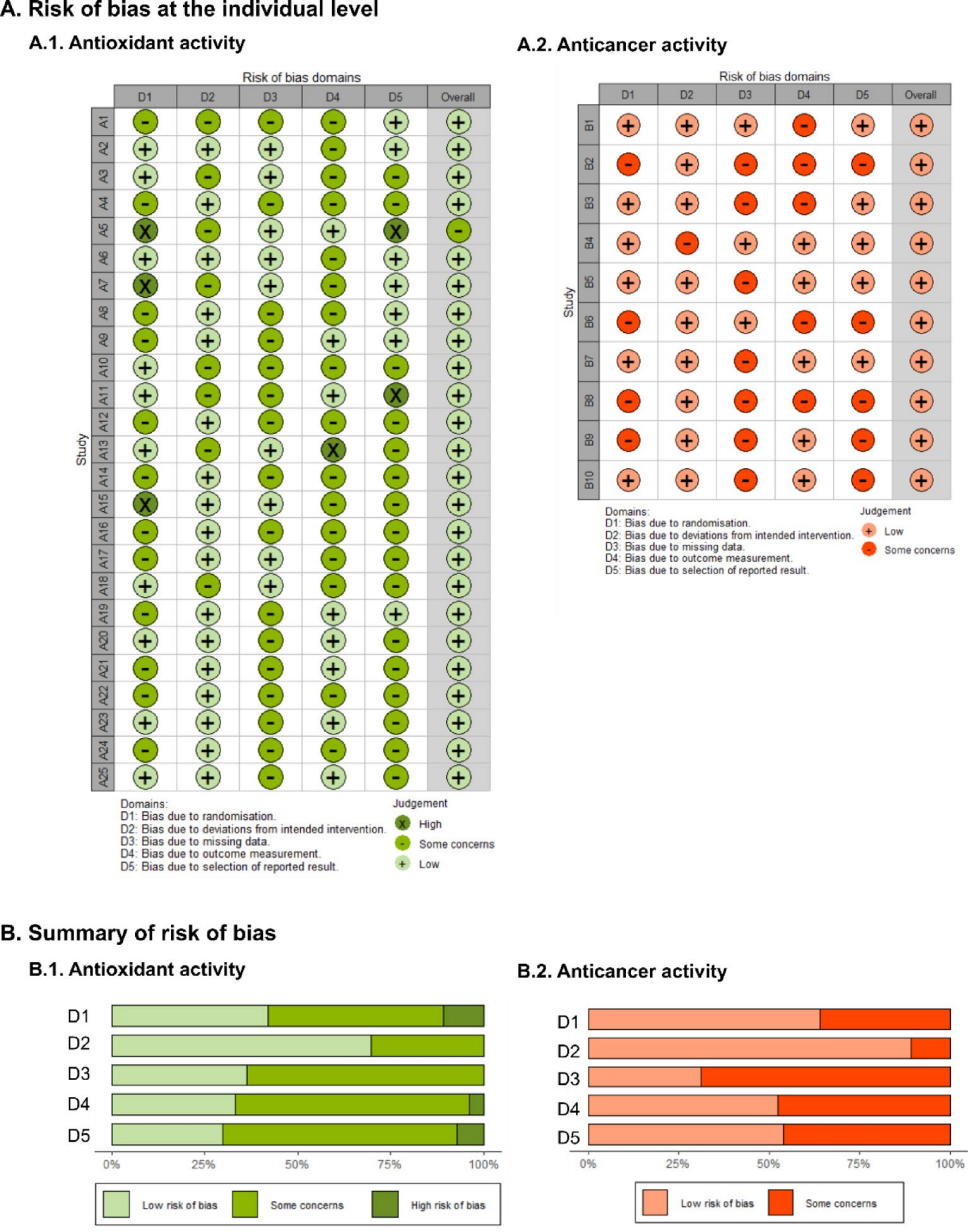
Risk of bias assessment: The risk of bias at the individual level (A) and summary of risk of bias (B). D1 is bias from randomization, D2 is bias from deviations from the intended intervention, D3 is bias due to missing data, D4 is bias from measurement outcomes, and D5 is bias from selecting reported results.

The determination of the risk of bias (ROB), including individual scores per article and overall summaries, was based on a detailed review of each article. The assessment considered various sources of bias: randomization in the sample (D1), the intended intervention (D2), missing data (D3), the measurement process of variables (D4), and the selection of reported outcome results (D5). The results of this risk of bias assessment for articles A1–A25 (studies on antioxidants) and B1–B10 (studies on anticancer agents) are illustrated in Fig. 2. Articles with overall high bias were excluded, and data extraction was not performed.

The selected articles served as the data source from which secondary data on antioxidants and anticancer activities were extracted. These data were then systematically organized into a spreadsheet, including the number of samples (sample size or n), average values (X̄), and standard deviations (SD) for both the control groups (C) and treatment (E) groups. The total number of studies addressing antioxidant and anticancer research or experiments was denoted as *k*.

Research from 24 articles on CMN antioxidants covered various aspects: citrus species, plant parts (leaf, juice, peel, pomace, and seed), nanoparticle types, nanoparticle sizes (10 to 1026 nm), synthesis methods [green synthesis (GSy), nanocomposite (NPC), nanoemulsion (NPE), and nanoparticle (NP)], antioxidant activity (measured the radical inhibition (%) and IC_50_ (µg/mL)), control (negative and positive), antioxidant assays, and dosages (in µg/mL; Table 2). The negative controls included silver nitrate (AgNO₃), citrus extract, sodium alginate, and pectin. The positive controls used were common antioxidant standards such as ascorbic acid (AsA), butylated hydroxytoluene (BHT), catechol, and 6-hydroxy-2,5,7,8-tetramethylchroman-2-carboxylic acid (Trolox). Antioxidant assays were used to measure the 2,2’-azino-bis (3-ethylbenzothiazoline-6-sulfonic acid) radical (ABTS), 2,2-diphenyl-1-picrylhydrazyl radical (DPPH), ferric reducing antioxidant power (FRAP), and hydroxyl radical (OH) contents. The CMN dose ranged from 15 to 50,000 µg/mL.

In Table 3, a summary of 10 studies on CMNs and their anticancer effects highlights various details. It included information on citrus species, plant parts (juice and peel), nanoparticle types and sizes (approximately 17.5 to 150 nm), synthesis methods (GSy), and anticancer activity (e.g., cancer cell growth inhibition (%) and IC_50_ (µg/mL)). It also included controls (both negative and positive), cancer cell lines (A-549, C6-Neural, DU-145, HeLa, HepG2, MCF-7, MDA-MB-468, melanoma, and SH-SY5Y), and dosages (in µg/mL). The cancer cell lines used in the study included A-549 (human lung cells, lung cancer), C6-Neural (rat brain cells, glioma or neural cancer), DU-145 (human prostate cells, prostate cancer), HeLa (human cervical cells, cervical cancer), HepG2 (human liver cells, liver cancer/hepatoblastoma), MCF-7 (human breast cells, hormone receptor-positive breast cancer), MDA-MB-468 (human breast cells, triple-negative breast cancer), Melanoma (human skin cells, melanoma/skin cancer), and SH-SY5Y (human neuroblastoma cells, sympathetic nervous system cancer) cells. The negative controls included untreated cancer cells and *Citrus limetta* carbon dots (CDOT), whereas doxorubicin (DOX) served as the positive control. The dosages ranged from 0.03 to 800 µg/mL.

The CMNs identified in the literature include silver nanoparticles with chitosan (Ag chitosan-NP), silver nanoparticles (Ag-NP), gold–iron oxide nanoparticle composite (Au@Fe₂O₃-NPC), gold nanoparticle (Au-NP), hydroxyapatite nanoparticle (Ca₁₀(PO₄)₆ (OH)₂-NP), cadmium oxide nanoparticle (CdO-NP), cerium dioxide nanoparticle (CeO₂-NP), cobalt nanoparticle (Co-NP), iron oxide nanoparticle (FeO-NP), hydrogel-based nanocomposite (Hydrogel-NPC), lime-derived nanoemulsion (Lime-NPE), lipid-based nanoemulsion (Lipid-NPE), luminescent nanoparticle (Luminescent-NP), nickel oxide nanoparticle (NiO-NP), pectin-based nanoemulsion (Pectin-NPE), tellurium nanoparticle (Te-NP), titanium dioxide nanoparticle (TiO₂-NP), vanadium nanoparticle (V-NP), whey-derived nanoemulsion (Whey-NPE), and zinc nanoparticle (Zn-NP).

**Table 2:** Recent studies on the use of citrus phytochemicals in the synthesis of nanocompounds with antioxidant activity.

No.	Species	Part	Nano	Size	Synthesis	Study	Control	Method	Dosage	References
A1	*Citrus limon*	Lf	Ag-NP	25.1	GSy	OXD and IC_50_	AgNO_3_, BHT, and EOC	DPPH	15–1000 µg/mL	^12^
A2	*Citrus aurantium*	Pe	V-NP	80	GSy	OXD	AAS	DPPH	250–2000 µg/mL	^13^
A3	*Citrus sinensis*	Pe	FeO-NP	22	GSy	OXD	-	ABTS	100 µg/mL	^24^
A4	*C. limon*, *C. limetta*, and *C. sinensis*	Pe	Ag-NP	27.5	GSy	OXD	BHT	DPPH	50–100 µg/mL	^25^
A5	*C. sinensis*	Pe	Ag-NP	71.8	GSy	OXD	AAS	DPPH	0.5 mL/3 mL	^26^
A6	*C. sinensis*	Pe	Lipid-NPE	245	NPE	IC_50_	AAS and Trolox	DPPH	-	^21^
A7	*Citrus reticulata*	Pe	Co-NP	18.45	GSy	OXD	BHT	DPPH, FRAP, and OH	200–1000 µg/mL	^27^
A8	*Citrus maxima*	Pe	Ag-NP	37.5	GSy	OXD	BHT	DPPH	0.1 mL/3.9 mL	^28^
A9	*Citrus latifolia*	Pc	Luminescent-NP	10	NPC	IC_50_	Quercetin	-	-	^29^
A10	*Citrus paradisi*	Se	Hydrogel-NPC	70	NPC	OXD	-	DPPH	0.0025-0.01 mL/mL	^30^
A11	*Citrus nobilis* x *Citrus deliciosa*	Pe	Ag-NP	399	GSy	OXD	EOC	DPPH	200 mg/mL	^31^
A12	*C. reticulata*	Pe	Pectin-NPE	230	NPE	OXD	EOC and Pectin	ABTS and DPPH	2–10 mg/mL	^32^
A13	*C. reticulata*	Pe	Whey-NPE	155–158	NPE	OXD	EOC	DPPH	0.1 µmol/L	^11^
A14	*C. sinensis*	Pe	Ag-NP	1026	GSy	OXD	AAS	DPPH	10–500 µg/mL	^33^
A15	*C. aurantium*	Pe	CeO_2_-NP	22.5	GSy	OXD	AAS	DPPH	10–100 µg/mL	^34^
A16	*C. limetta*	Pc	Ag-NP	20	GSy	OXD	EOC	DPPH	10–100 µL	^35^
A17	*C. limetta*	Pe	TiO_2_-NP	12.5	GSy	OXD	NaAlg	DPPH	0.05 g/mL	^36^
A18	*C. aurantium*	Pe	Zn-NP	41.2	GSy	IC_50_	BHT	DPPH	-	^37^
A19	*C. limon*	Pe	Lime-NPE	-	NPE	IC_50_	AAS	DPPH	-	^38^
A20	*C. aurantium*	Pc	NiO-NP	75.5	GSy	OXD	BHT	DPPH, FRAP, and OH	20–100 µg/mL	^39^
A21	*C. limetta*	Pe	CdO-NP	51.5	GSy	OXD	AAS	ABTS and DPPH	20–100 µg/mL	^40^
A22	*C. limon*	Jc	Ag chitosan-NP	20	GSy	OXD	AAS	DPPH	20–100 µg/mL	^41^
A23	*C. clementina*	Pe	Ag-NP	17.5	GSy	OXD	AAS, Catechol, and EOC	ABTS and DPPH	20–100 µg/mL	^42^
A24	*C. sinensis*	Jc	Au@Fe_2_O_3_-NPC		GSy	OXD	AAS	DPPH	30–1000 µg/mL	^43^
A25	*C. limetta*	Pe	Au-NP	64	GSy	OXD	Trolox	ABTS, DPPH, NO, and OH	20–100 µg/mL	^20^

Part refers to the extracted parts of the citrus plant, including the leaf (Lf), juice (Jc), peel (Pe), pomace (Pc), and seed (Se) parts, and the size of the nanoparticles in nanometers (nm).

AAS, ascorbic acid; ABTS, 2,2’-azino-bis (3-ethylbenzothiazoline-6-sulfonic acid) radical for the ABTS assay; BHT, butylated hydroxytoluene; DPPH, 2,2-diphenyl-1-picrylhydrazyl radical for the DPPH assay; EOC, extracted citrus phytochemical; FRAP, ferric reducing antioxidant power; GSy, green synthesized; NaAlg, sodium alginate; NO, nitric oxide radical; NP, nanoparticle; NPC, nanocomposite; NPE, nanoemulsion; OH, hydroxyl radical; OXD, antioxidant capacity (%); Ref. reference; Trolox, 6-hydroxy-2,5,7,8-tetramethylchroman-2-carboxylic acid.

*Nanoparticle*: Ag chitosan-NP, silver nanoparticles with chitosan; Ag-NP, silver nanoparticle; Au@Fe₂O₃-NPC, gold–iron oxide nanoparticle composite; Au-NP, gold nanoparticle; CdO-NP, cadmium oxide nanoparticle; CeO₂-NP, cerium dioxide nanoparticle; Co-NP, cobalt nanoparticle; FeO-NP, iron oxide nanoparticle; Hydrogel-NPC, hydrogel-based nanocomposite; Lime-NPE, lime-derived nanoemulsion; Lipid-NPE, lipid-based nanoemulsion; Luminescent-NP, luminescent nanoparticle; NiO-NP, nickel oxide nanoparticle; Pectin-NPE, pectin-based nanoemulsion; TiO₂-NP, titanium dioxide nanoparticle; V-NP, vanadium nanoparticle; Whey-NPE, whey-derived nanoemulsion; Zn-NP, zinc nanoparticle.

**Table 3:** Studies on the engineering of nanoparticles based on citrus phytochemicals for anticancer applications.

No	Species	Part	Nano	Size	Synthesis	Study	Control	Cell line	Dosage	Ref.
B1	*C. aurantium*	Pe	V-NP	80	GSy	CVB and IC_50_	DOX	MCF-7	1.56–100 µg/mL	^13^
B2	*C. limon*	Jc	Ca₁₀(PO₄) ₆(OH)₂-NP	30	GSy	CVB	UCC	SH-SY5Y	5–800 µg/mL	^14^
B3	*Citrus aurantiifolia*, *C. aurantium*, and *C. limon*	Jc	Te-NP	101–150	GSy	CVB and IC_50_	UCC	Melanoma	5–100 µg/mL	^15^
B4	*C. limetta*	Pe	Ag-NP	10.5	GSy	IC_50_	CDOT	HeLa and MCF-7	5–200 µg/mL	^44^
B5	*C. sinensis*	Pe	Ag-NP	30	GSy	CVB	UCC	DU-145	-	^45^
B6	*C. aurantium*	Pe	CeO_2_-NP	22.5	GSy	CVB	UCC	HeLa	10–125 µg/mL	^34^
B7	*Citrus unshiu*	Pe	Ag-NP	23	GSy	IC_50_	DOX	A-549	-	^46^
B8	*Citrus macroptera*	Jc	Au-NP	-	GSy	CVB and IC_50_	UCC	A-549, HepG2, and MDA-MB-468	0.03–0.3 µg/mL	^16^
B9	*C. limetta*	Pe	CdO-NP	46	GSy	CVB and IC_50_	UCC and DOX	A-549	10–320 µg/mL	^40^
B10	*Citrus clementina*	Pe	Ag-NP	17.5	GSy	CVB and IC_50_	UCC and DOX	C6-Neural	20–200 µg/mL	^42^

Part refers to the extracted parts of the citrus plant, including juice (Jc) and peel (Pe), and the size of the nanoparticles in nanometers (nm).

A-549, human lung cancer cell line; C6-Neural, murine glioma cancer cell line; CDOT, carbon dot of *C. limetta*; CVB, cancer cell viability (%); DOX, doxorubicin; DU-145, human prostate cancer cell line; EOC, extracted citrus phytochemical; GSy, green synthesized; HeLa, a human cervical cancer cell line; HepG2, human hepatoma cell line; MCF-7, Michigan Cancer Foundation-7 (a human breast cancer cell line); MDA-MB-468, human breast cancer cell line; Melanoma, a type of skin cancer that originates from melanocytes; NaAlg, sodium alginate; NP, nanoparticle; Ref. reference; SH-SY5Y, a human neuroblastoma cancer cell line; UCC, untreated cancer cell.

Ag-NP, silver nanoparticles; Au-NP, gold nanoparticle; Ca₁₀(PO₄) ₆(OH)₂-NP, hydroxyapatite nanoparticle; CdO-NP, cadmium oxide nanoparticle; CeO₂-NP, cerium dioxide nanoparticle; Te-NP, tellurium nanoparticle; V-NP, vanadium nanoparticle.

### Analysis and validation of a meta-analysis

The meta-analysis conducted was based on the methodology introduced by Marín-Martínez and Sánchez-Meca (2010), utilizing Hedges’ effect size (d) calculations^18^. Hedges’ method quantifies the difference between treatment and control groups by dividing the mean difference of both averages by the pooled standard deviation of both groups (Eq. 1). Equation 2 provides the formula for computing the pooled standard deviation (SD pooled), with adjustments made for small sample sizes (5–20 units per treatment) via Eq. 3 or Hedges’ correction factor (J). The combined effect size (d_++_) was determined via Eq. 4, where a random effects model was used to estimate the variance of heterogeneity and assess its impact on dependent variables such as antioxidant and anticancer activities. The value of d_++_indicates a larger effect size (> 1) and a small effect size (< 0.8)^47^. The variation in Hedges’ (Vd_++_) is calculated via Eq. 5. Sources of heterogeneity include study types (measured by IC_50_ in µg/mL, radical inhibition (%), and cell viability in %), citrus species, plant parts, nanoparticle characteristics [type and size (1–50, 51–100, 101–500, or > 500 nm)], coating materials, control groups (negative and positive), cancer cell lines, and types of cancer. Heterogeneity levels are evaluated via the Q statistic and I^2^ statistic (Eq. 6); significant heterogeneity is indicated by *P* < 0.05 or I^2^ > 50%^48^. High heterogeneity necessitates d_++_calculations for each source or subgroup analysis in the meta-analysis. The validation metrics for meta-analysis models involve the use of the fail-safe N (FsN) calculation, as defined by Eq. 7^49^. The model is deemed robust (Rb.) if the computed FsN ≥ FsN model (5k + 10), where k denotes the number of studies in the meta-analysis. Conversely, if the computed FsN is less than the FsN model, the meta-analysis model is considered not robust (NRb.).

1 $$\:d = \:\frac{{\overline{{\text{X}}} E - \overline{{\text{X}}} C}}{{{\text{SD}}\:_{{pooled}} }}$$

2 $$\:{\text{S}\text{D}\:}_{pooled}=\sqrt{\frac{\left(nE-1\right){\left(\text{S}\text{D}\text{E}\right)}^{2}+\left(nC-1\right){\left(\text{S}\text{D}\text{C}\right)}^{2}}{\text{n}\text{E}+nC-2}\times\:J}$$

3 $$\:J=1-\:\frac{3}{4\left(nE+\:nC\right)-9}$$

4 $$\:{d}_{++}=\frac{{\sum\:}_{i=1}^{n}{W}_{i}{d}_{1}}{{\sum\:}_{i=1}^{n}{W}_{i}}$$

5 $$\:{Vd}_{++}=\frac{nC+nE}{nC.nE}+\frac{{d}^{2}}{2\left(nC+nE\right)}$$

6 $$\:{I}^{2}=\frac{Q-df}{Q}\times\:100\%$$

7 $$\:Fail-safe\:N=\left(\frac{{\left(\frac{\varSigma\:Z}{1.64}\right)}^{2}-k}{{1.64}^{2}-k}\right)$$

The symbols used for Eqs. 1–6 are as follows: X̄E denotes the average of the treatment group, whereas X̄C represents the mean of the control group. The _pooled_ SDs represent the combined standard deviation of both the treatment and control groups. Thus, nE and nC refer to the respective sample sizes of the treatment and control groups. Additionally, SDE and SDC indicate the standard deviations of the treatment and control groups, respectively. Furthermore, J served as the correction factor for small sample sizes, Wi represented the weight assigned to each study within the meta-analysis, Q measured the Q statistic for assessing heterogeneity, and df represented the degrees of freedom associated with the Q statistic. Z represents the total z score from each *k*calculated within Hedges’. The calculation of Hedges’ and FsN was conducted via OpenMEE, version 2016^5^.

Meta-network analysis is used to rank CMNs based on citrus species and types of nanoparticles for antibacterial and anticancer activities. The results for antioxidant and anticancer activities, which show significant findings for the species and nanoparticle subgroups, were selected for ranking via network meta-analysis. Additionally, a trend analysis (using a meta-regression approach) of the effects of the dosage of CMN on the antioxidant and anticancer activities of citrus species and types of nanoparticles was conducted. The meta-network analysis and meta-regression analysis were conducted via R software version 4.3.2 with the primary packages “lme4” version 1.1–35 and “multinma” version 0.2.1^51^.

## Results

### Antioxidant activity

Table 4 presents the results of the meta-analysis of CMNs. Overall, CMNs did not exhibit statistically significant antioxidant activity, as reflected by the small effect sizes observed for general antioxidant capacity (d_++_ = 0.2; *P* = 0.309) and radical scavenging activity (d_++_ = 0.1; *P* = 0.602). In contrast, the IC_50_-based assay demonstrated a significant and robust effect (d_++_ = 3.49; *P* < 0.05), indicating strong antioxidant potential under this specific measurement.

**Table 4:** Antioxidant and anticancer activities of citrus-mediated nanoformulations.

	k	Effect size		Heterogeneity		FsN
		d_++_ ± SE	*P* value	I^2^	*P* value	
Antioxidant						
Overall value	206	0.2 ± 0.2^NRb^.	0.309	82.2	< 0.001	540
IC_50_ (µg/mL)	9	3.49 ± 1.41^Rb^.	0.013	86.2	< 0.001	114
Radical inhibition (%)	197	0.1 ± 0.2^NRb^.	0.602	81.9	< 0.001	204
Anticancer						
Overall value	143	−3.05 ± 0.28^Rb^.	< 0.001	79.9	< 0.001	24,819
IC_50_ (µg/mL)	14	8.77 ± 3.57^Rb^.	0.014	91.2	< 0.001	162
Cancer cell viability (%)	129	−3.26 ± 0.25^Rb^.	< 0.001	75.8	< 0.001	29,195

The FsN value is considered robust (Rb.) if FsN ≥ 5k + 10, otherwise not robust (NRb.).

d_++_, Hedges’ mean difference; FsN, fail-safe N; I^2^heterogeneity derived from Cochran’s value; N, number of studies; SE, standard error.

Table 5 presents the results of the subgroup meta-analysis of IC_50_ antioxidant values. The analysis revealed that citrus peel (plant part subgroup) and polyvinyl alcohol (coating material subgroup) had significant and substantial effect sizes (d_++_ >1; *P* < 0.05). In comparison, the other subgroups, which included species, nanoparticle type, particle size, and control type, did not have statistically significant effects.

**Table 5:** Antioxidant IC_50_ (µg/mL) values of citrus-mediated nanoformulations by citrus species, plant parts, nanoparticle types, sizes, coatings, and control types.

	K	Effect size		Heterogeneity		FsN
		d_++_ ± SE	*P* value	I^2^	*P* value	
Species						
*C. aurantium*	1	2.86 ± 1.16^NRb^.	-	-	-	2
*C. latifolia*	2	2.88 ± 2.58^NRb^.	0.265	89.7	0.002	14
*C. limon*	4	1.93 ± 2.24^NRb^.	0.39	87.9	< 0.001	5
*C. sinensis*	2	5 ± 4.52^NRb^.	0.268	88.2	0.004	6
Plant parts						
Leaf	2	−0.77 ± 0.63^NRb^.	0.22	5.28	0.304	-
Peel	5	5.1 ± 2.13^Rb^.	0.016	84.1	< 0.001	63
Pomace	2	2.88 ± 2.58^NRb^.	0.265	89.7	0.002	14
Nanoparticle types						
Ag-NP	2	−0.77 ± 0.63^NRb^.	0.22	5.28	0.304	-
Lime-NPE	2	4.67 ± 4.4^NRb^.	0.289	90.3	0.001	2
Lipid-NPE	2	5 ± 4.52^NRb^.	0.268	88.2	0.004	6
Luminescent-NPC	2	2.88 ± 2.58^NRb^.	0.265	89.7	0.002	14
Zn-NP	1	2.86 ± 1.16^NRb^.	-	-	-	-
Sizes (nm)						
1–50	5	1.99 ± 1.65^NRb^.	0.229	86.2	< 0.001	14
101–500	4	7.19 ± 3.76^Rb^.	0.056	88	< 0.001	42
Coatings						
Citrus extract	3	0.29 ± 1.15^NRb^.	0.803	77	0.013	-
Lipid	2	5 ± 4.52^NRb^.	0.268	88.2	0.004	6
Polyvinyl alcohol	4	1.37 ± 0.54^Rb^.	0.011	85.2	< 0.001	61
Control types						
Ascorbic acid	3	1.17 ± 5.6^Rb^.	0.037	81.6	0.004	34
BHT	2	1.22 ± 1.53^NRb^.	0.427	78.6	0.031	-
*C. limon*	1	−1.47 ± 0.92^NRb^.	-	-	-	-
Quercetin	2	2.88 ± 2.58^NRb^.	0.265	89.7	0.002	14
Trolox	1	0.91 ± 0.86^NRb^.	-	-	-	-

The FsN value is considered robust (Rb.) if FsN ≥ 5k + 10, otherwise not robust (NRb.).

BHT, butylated hydroxytoluene; d_++_, Hedges’ mean difference; FsN, fail-safe N; I^2^ heterogeneity derived from Cochran’s value; N, number of studies; NP, nanoparticle; NPC, nanocomposite; NPE, nanoemulsion; SE, standard error; Trolox, 6-hydroxy-2,5,7,8-tetramethylchroman-2-carboxylic acid.

Table 6 presents the subgroup analysis for radical inhibition, while Fig. 4 illustrates the percentage of radical inhibition across various citrus species. Species such as *C. limetta*, *C*. *limon*, *C*. *maxima*, *C*. *paradisi*, *C*. *reticulata*, and *C*. *sinensis* presented strong effect sizes (|d_++_| > 0.8; *P* < 0.05). Notably, *C*. *limetta*, *C*. *maxima*, and *C*. *sinensis* presented negative effect sizes, indicating that the treatment groups presented lower values than did their respective controls. The models involving *C*. *limetta*, *C*. *limon*, *C*. *paradisi*, and *C*. *sinensis* remained statistically robust. Within the plant-based subgroup, the leaf, pomace, and seed samples derived from citrus sources presented significant and robust effect sizes (|d_++_| > 0.8; *P* < 0.05). Among these, pomace had a negative effect size, implying reduced radical inhibition relative to that in the control groups. For the nanoparticle subgroup, all categories showed statistically significant results, each with a strong effect size (|d_++_| > 0.8; *P* < 0.05). The nanoparticles that yielded positive effect sizes included Ag-NPs, Ag chitosan-NPs, CeO₂-NPs, hydrogel-NPC, pectin-NPE, TiO₂-NPs, and whey-NPE. In contrast, Au-NPs, Au@Fe₂O₃-NPC, CdO-NPs, Co-NPs, Fe₃O₄-NPs, NiO-NPs, and V-NPs presented negative values. However, the model involving Fe₃O₄-NPs lacked robustness. The nanoparticle size also influenced the efficacy. Particles within the 101–500 nm range produced the greatest effect size (d_++_ = 3.44), surpassing those in the 1–50 nm (d_++_ =0.77), 51–100 nm (d_++_ = −1.56), and > 500 nm (d_++_ = −13.7) ranges (*P* < 0.01). Subgroup analysis further indicated that coating materials such as citrus extract, hydroxypropyl methylcellulose, pectin, and whey significantly enhanced radical inhibition (*P* < 0.01). All models were robust, except for citrus extract, which, although statistically strong, exhibited an exceptionally high positive effect size. Finally, the meta-analysis based on the control types used in the radical inhibition assays revealed a clear contrast. The positive controls (including AsA, BHT, catechol, and Trolox) had strong negative effects (|d_++_| >1; *P* < 0.001), reflecting greater antioxidant capacity than other treatments did. Conversely, negative controls (such as AgNO₃, *C*. *clementina*, *C*. *limon*, *C*. *reticulata*, *C*. *unshiu*, hydrogel films, pectin, and sodium alginate) yielded positive effect sizes (*P* < 0.05), indicating lower radical inhibition compared to treatments.

**Table 6:** Radical Inhibition (%) by citrus species, plant parts, nanoparticle types, sizes, coatings, and control types.

	k	Effect size		Heterogeneity		FsN
		d_++_ ± SE	*P* value	I^2^	*P* value	
Species						
*C. aurantium*	29	−0.77 ± 0.4^Rb^.	0.055	74.9	< 0.001	270
*C. clementina*	20	0.55 ± 0.64^NRb^.	0.392	83.7	< 0.001	-
*C. limetta*	51	−0.87 ± 0.28^Rb^.	0.002	71.8	< 0.001	661
*C. limon*	26	2.25 ± 0.33^Rb^.	< 0.001	55.5	< 0.001	993
*C. maxima*	2	−1.53 ± 0.66^NRb^.	0.02	-	0.635	3
*C. nobilis* × *C. deliciosa*	1	1.93 ± 0.99^NRb^.	-	-	-	1
*C. paradisi*	3	3.05 ± 1.26^Rb^.	0.015	82.5	0.003	37
*C. reticulata*	48	1.03 ± 0.46^NRb^.	0.025	84.4	< 0.001	190
*C. sinensis*	15	−6.57 ± 1.55^Rb^.	< 0.001	87.6	< 0.001	377
Plant parts						
Juice	11	−2.2 ± 2.01^NRb^.	0.273	90.7	< 0.001	-
Leaf	21	1.76 ± 0.3^Rb^.	< 0.001	42.8	0.02	496
Peel	139	−0.02 ± 0.25^NRb^.	0.952	82.7	< 0.001	78
Pomace	23	−0.93 ± 0.23^Rb^.	< 0.001	31	0.08	206
Seed	3	3.05 ± 1.26^Rb^.	0.015	82.5	0.003	37
Nanoparticle types						
Ag-NP	59	1.04 ± 0.37^Rb^.	0.005	81.7	< 0.001	681
Ag chitosan-NP	5	4.92 ± 0.75^Rb^.	< 0.001	-	0.63	80
Au-NP	30	−1.11 ± 0.16^Rb^.	< 0.001	-	0.92	528
Au@Fe_2_O_3_-NPC	6	−8.95 ± 2.02^Rb^.	< 0.001	70.9	0.004	132
CdO-NP	10	−8.4 ± 1.76^Rb^.	< 0.001	87.5	< 0.001	235
CeO_2_-NP	5	2.47 ± 0.65^Rb^.	< 0.001	37.9	0.169	43
Co-NP	20	−2.42 ± 0.39^Rb^.	< 0.001	55.9	0.001	649
Fe_3_O_4_-NP	1	−0.86 ± 0.85^NRb^.	-	-	-	-
Hydrogel-NPC	3	3.05 ± 1.26^Rb^.	0.015	82.5	0.003	37
NiO-NP	20	−1.14 ± 0.23^Rb^.	< 0.001	18.6	0.223	237
Pectin-NPE	20	3.85 ± 0.62^Rb^.	< 0.001	75.8	< 0.001	817
TiO_2_-NP	6	2.06 ± 0.42^Rb^.	< 0.001	-	0.913	51
V-NP	4	−6.16 ± 2.78^Rb^.	0.027	90.4	< 0.001	63
Whey-NPE	8	4.04 ± 1^Rb^.	< 0.001	73	< 0.001	144
Sizes (nm)						
1–50	90	0.77 ± 0.28^Rb^.	0.006	81.4	< 0.001	989
51–100	71	−1.56 ± 0.23^Rb^.	< 0.001	68.2	< 0.001	4,062
101–500	31	3.44 ± 0.544^Rb^.	< 0.001	79.2	< 0.001	1,437
> 500	6	−1.37 ± 0.303^Rb^.	< 0.001	63.6	< 0.001	100
Coatings						
Citrus extract	166	−0.52 ± 0.2^Rb^.	0.009	80.2	< 0.001	2,422
Hydroxy propyl methylcellulose	3	3.05 ± 1.26^Rb^.	0.015	82.5	0.003	37
Pectin	20	3.85 ± 0.62^Rb^.	< 0.001	75.8	< 0.001	817
Whey	8	4.04 ± 1^Rb^.	< 0.001	73.9	< 0.001	144
Control types						
AgNO_3_	7	4.19 ± 0.55^Rb^.	< 0.001	-	1	143
Ascorbic acid	44	−3.1 ± 0.73^Rb^.	< 0.001	88	< 0.001	1,023
BHT	56	−1.13 ± 0.26^Rb^.	< 0.001	70.6	< 0.001	810
Catechol	5	−2.15 ± 0.47^Rb^.	< 0.001	-	0.491	38
*C. clementina*	10	3.19 ± 0.41^Rb^.	< 0.001	1.46	0.425	247
*C. limetta*	3	0.42 ± 0.48^NRb^.	0.384	-	0.972	-
*C. limon*	7	1.72 ± 0.36^Rb^.	< 0.001	-	0.999	52
*C. nobilis* × *C. deliciosa*	1	1.93 ± 0.99^NRb^.	-	-	-	1
*C. reticulata*	8	4.04 ± 1^Rb^.	< 0.001	73.9	< 0.001	144
*C. unshiu*	10	3.73 ± 0.86^Rb^.	< 0.001	74.7	< 0.001	201
Hydrogel films	3	3.05 ± 1.26^Rb^.	0.015	82.5	0.003	37
Pectin	10	4.12 ± 0.98^Rb^.	< 0.001	79.1	< 0.001	198
Sodium alginate	3	1.74 ± 0.55^NRb^.	0.002	-	0.949	9
Trolox	30	−1.11 ± 0.16^Rb^.	< 0.001	-	0.92	528

The FsN value is considered robust (Rb.) if FsN ≥ 5k + 10, otherwise not robust (NRb.).

BHT, butylated hydroxytoluene; d_++_, Hedges’ mean difference; FsN, fail-safe N; I^2^ heterogeneity derived from Cochran’s value; N, number of studies; NP, nanoparticle; NPC, nanocomposite; NPE, nanoemulsion; SE, standard error; Trolox, 6-hydroxy-2,5,7,8-tetramethylchroman-2-carboxylic acid.

### Anticancer activity

The anticancer activity of CMNs had strong and significant effects on overall outcomes, including IC_50_ values and a reduction in cancer cell viability (|d_++_| >1; *P* < 0.05; Table 4). Notably, only the IC50 value for cancer inhibition positively influenced both the reduction in cancer cell viability and the overall effect size. This likely reflects the fundamental nature of the cell viability data, which typically indicates decreased viability in cancer cells treated with CMNs compared with untreated control groups (usually cancer cells without any intervention). Accordingly, the administration of therapeutic agents such as CMNs is expected to reduce cancer cell viability.

In the evaluation of 50% inhibition of cancer cells, several effect sizes were observed within the subgroup meta-analysis (Table 7). The species group, specifically *C*. *macroptera*, exhibited a robust and positive effect size (d_++_ = 2.25; *P* < 0.001). Additionally, plant parts such as juice presented a similarly robust effect size of 2.26 (*P* < 0.001). Subgroups involving nanoparticles, namely, Au-NPs and Te-NPs, also yielded significant results (|d_++_| >1; *P* < 0.001; Rb.). Furthermore, nanoparticles with sizes between 101 and 500 nm demonstrated an effect size of 2.26 (*P* < 0.001; Rb.). Citrus extract, employed as the sole coating, had a significant effect size (|d_++_| >1; *P* < 0.05). The positive control groups treated with DOX presented a notable effect size (d_++_ = 1.4; *P* < 0.001), whereas the negative control groups treated with CDOT and UCC presented divergent results (|d_++_| >1, *P* < 0.05; Rb.). An examination of various cancer cell lines and types revealed that cervical cancer (HeLa), lung cancer (A-549), and skin cancer (melanoma) all had effect sizes > 1 and were statistically significant (*P* < 0.01). Notably, only HeLa cells exhibited a negative effect size with a non-robust model.

**Table 7:** Anticancer IC_50_ (µg/mL) values of citrus-mediated nanoformulations by citrus species, plant parts, nanoparticle types, sizes, coating, control types, cancer cell lines, and cancer types.

	k	Effect size		Heterogeneity		FsN
		d_++_ ± SE	*P* value	I^2^	*P* Value	
Species						
*C. aurantiifolia*	1	2.26 ± 0.66^NRb^.	-	-	-	4
*C. aurantium*	2	1.27 ± 0.87^NRb^.	0.144	85	0.01	14
*C. clementina*	1	1.22 ± 0.36^NRb^.	-	-	-	4
*C. limetta*	5	−1.01 ± 0.71^NRb^.	0.158	90.9	< 0.001	23
*C. limon*	1	2.26 ± 0.66^NRb^.	-	-	-	4
*C. macroptera*	3	2.25 ± 0.38^Rb^.	< 0.001	-	0.999	37
*C. unshiu*	1	1.16 ± 0.33^NRb^.	-	-	-	4
Plant parts						
Juice	6	2.26 ± 0.27^Rb^.	< 0.001	-	1	152
Peel	8	−1.71 ± 4.1^NRb^.	0.676	91.6	< 0.001	-
Nanoparticles						
CdO-NP	1	1.93 ± 0.56^NRb^.	-	-	-	4
Au-NP	3	2.25 ± 0.38^Rb^.	< 0.001	-	0.999	37
Ag-NP	6	−6.73 ± 6.68^NRb^.	0.314	91.3	< 0.001	5
Te-NP	3	2.26 ± 0.38^Rb^.	< 0.001	-	1	37
V-NP	1	5.06 ± 1.67^NRb^.	-	-	-	3
Sizes (nm)						
1–50	10	5.56 ± 5.37^NRb^.	0.3	91.8	< 0.001	17
51–100	1	5.06 ± 1.67^NRb^.	-	-	-	3
101–500	3	2.26 ± 0.38^Rb^.	< 0.001	-	1	37
Coating						
Citrus extract	14	8.77 ± 3.57^Rb^.	0.014	91.2	< 0.001	162
Control types						
CDOT	4	−1.8 ± 0.78^Rb^.	0.025	89.6	< 0.001	51
Doxorubicin	4	1.4 ± 0.58^Rb^.	0.012	83.9	< 0.001	62
UCC	6	2.3 ± 0.27^Rb^.	< 0.001	-	1	152
Cancer cell lines						
A-549	3	2.9 ± 0.11^Rb^.	0.007	75.3	0.017	37
C6-Neural	1	1.2 ± 0.4^NRb^.	-	-	-	4
HeLa	2	−2.4 ± 0.5^NRb^.	< 0.001	-	0.444	16
HepG2	1	2.3 ± 0.66^NRb^.	-	-	-	4
MCF-7	3	−3.2 ± 0.4^NRb^.	0.428	91.7	< 0.001	-
MDA-MB-468	1	2.2 ± 0.65^NRb^.	-	-	-	4
Melanoma	3	2.3 ± 0.38^Rb^.	< 0.001	-	1	37
Cancer types						
Breast	4	1.1 ± 0.42^NRb^.	0.806	91.7	< 0.001	-
Cervix	2	−2.4 ± 5^NRb^.	< 0.001	-	0.444	16
Liver	1	2.3 ± 0.6^NRb^.	-	-	-	4
Lung	3	2.9 ± 1.1^Rb^.	0.007	75.3	0.018	37
Nerve	1	1.2 ± 0.36^NRb^.	-	-	-	4
Skin	3	2.3 ± 0.38^Rb^.	< 0.001	-	1	37

The FsN value is considered robust (Rb.) if FsN ≥ 5k + 10, otherwise not robust (NRb.).

A-549, human lung cancer cell line; C6-Neural, murine glioma cancer cell line; CDOT, carbon dot of *C. limetta*; d_++_, Hedges’ mean difference; FsN, fail-safe N; HeLa, a human cervical cancer cell line; HepG2, human hepatoma cell line; I^2^heterogeneity derived from Cochran’s value; MCF-7, Michigan Cancer Foundation-7 (a human breast cancer cell line); MDA-MB-468, human breast cancer cell line; Melanoma, a type of skin cancer that originates from melanocytes; N, number of studies; NP, nanoparticle; NPC, nanocomposite; NPE, nanoemulsion; SE, standard error; UCC, untreated cancer cell.

Table 8 summarizes the subgroup meta-analysis results regarding the effects of citrus phytochemicals in nanoparticle form on cancer cell inhibition. Subgroups categorized by citrus species, such as *C*. *aurantiifolia*, *C*. *clementina*, *C*. *limetta*, *C*. *limon*, *C*. *macroptera*, and *C*. *sinensis*, presented significant negative effect sizes (|d_++_| >1; *P* < 0.001; Rb.). Similar findings were observed across subgroups based on plant part and type of nanoparticle. Specifically, both citrus juice and peel had notable negative effects (|d_++_| >1; *P* < 0.001; Rb.). Moreover, the Ag-NPs, Au-NPs, CdO-NPs, CeO2-NPs, Ca_10_(PO_4_)_6_(OH)_2_-NPs, and Te-NPs generally had negative effect sizes, except V-NPs, which had a positive effect size (|d_++_| >1; *P* < 0.05; Rb.). In terms of the CMNs’ size, those ranging from 1 to 50 nm and 101–500 nm presented negative effects, whereas those ranging from 51 to 100 nm presented positive effects (|d_++_| >1; *P* < 0.001; Rb.). Additionally, the citrus extract used as a coating yielded an effect size of −3.26 (*P* < 0.001; Rb.). The positive control (DOX) had an effect size of 5.54, whereas the negative controls CDOT and UCC had effect sizes of −3.4 and − 3.61, respectively (*P* < 0.001; Rb.). Finally, various cancer cell types, such as A-549 (lung cancer), C6-neural and SH-SY5Y (nerve cancer), DU-145 (prostate cancer), HeLa (cervix cancer), HepG2 (lung cancer), MDA-MB-468 (breast cancer), and melanoma (skin cancer), manifested negative effect sizes (|d_++_| >1; *P* < 0.05). Notably, only the SH-SY5Y cell line exhibited a less robust meta-analysis model.

**Table 8:** Inhibition of cancer cells (%) by citrus-mediated nanoformulations across citrus species, plant parts, types, sizes, coatings, controls, cancer cell lines, and cancer types.

	k	Effect size		Heterogeneity		FsN
		d_++_ ± SE	*P* value	I^2^	*P* value	
Species						
*C. aurantiifolia*	14	−5.07 ± 0.55^Rb^.	< 0.001	27.1	0.164	651
*C. aurantium*	27	−0.86 ± 0.65^Rb^.	0.186	83.7	< 0.001	198
*C. clementina*	8	−1.1 ± 0.24^Rb^.	< 0.001	75.8	< 0.001	222
*C. limetta*	34	−3.43 ± 0.38^Rb^.	< 0.001	63.5	< 0.001	2,602
*C. limon*	24	−2.51 ± 0.38^Rb^.	< 0.001	61.4	< 0.001	906
*C. macroptera*	18	−3.81 ± 0.68^Rb^.	< 0.001	77.2	< 0.001	641
*C. sinensis*	4	−6.89 ± 1.1^Rb^.	< 0.001	-	0.498	58
Plant parts						
Juice	70	−2.95 ± 0.25^Rb^.	< 0.001	64	< 0.001	9,573
Peel	59	−3.7 ± 0.51^Rb^.	< 0.001	82.8	< 0.001	5,277
Nanoparticles						
Ag-NP	40	−4.35 ± 0.46^Rb^.	< 0.001	72.9	< 0.001	4,135
Au-NP	18	−3.81 ± 0.68^Rb^.	< 0.001	77.2	< 0.001	641
CdO-NP	6	−3.64 ± 0.83^Rb^.	< 0.001	52.1	0.064	89
CeO_2_-NP	6	−1.46 ± 0.37^Rb^.	< 0.001	83.3	< 0.001	137
Ca_10_(PO_4_)_6_(OH)_2_-NP	10	−1.02 ± 0.43^NRb^.	0.01913	51.9	0.028	40
Te-NP	42	−3.13 ± 0.24^Rb^.	< 0.001	32.6	0.024	4,251
V-NP	7	5.54 ± 0.7^Rb^.	< 0.001	-	0.609	168
Sizes (nm)						
1–50	79	−3.92 ± 0.33^Rb^.	< 0.001	75.7	< 0.001	13,932
51–100	7	5.54 ± 0.7^Rb^.	< 0.001	-	0.609	168
101–500	43	−3.07 ± 0.25^Rb^.	< 0.001	37.1	0.009	4,325
Coating						
Citrus extract	129	−3.26 ± 0.25^Rb^.	< 0.001	75.8	< 0.001	29,195
Control types						
CDOT	28	−3.4 ± 0.43^Rb^.	< 0.001	65.77	< 0.001	1,706
Doxorubicin	7	5.54 ± 0.7^Rb^.	< 0.001	-	0.609	168
UCC	94	−3.61 ± 0.27^Rb^.	< 0.001	70.75	< 0.001	20,302
Cancer cell lines						
A-549	12	−4.14 ± 0.71^Rb^.	< 0.001	62.85	0.002	382
C6-Neural	8	−1.1 ± 0.24^Rb^.	< 0.001	83.9	< 0.001	222
DU-145	4	−6.89 ± 1.1^Rb^.	< 0.001	-	0.498	58
HeLa	20	−4.43 ± 0.68^Rb^.	< 0.001	71.9	< 0.001	1,107
HepG2	6	−4.03 ± 1.37^Rb^.	0.003	81.8	< 0.001	59
MCF-7	21	−0.77 ± 0.88^NRb^.	0.379	87.1	< 0.001	26
MDA-MB-468	6	−2.99 ± 1.08^Rb^.	0.006	77	< 0.001	50
Melanoma	42	−3.13 ± 0.24^Rb^.	< 0.001	32.6	0.024	4,251
SH-SY5Y	10	−1.02 ± 0.43^NRb^.	0.019	51.9	0.028	40
Cancer types						
Breast	27	−1.38 ± 0.7^Rb^.	0.048	85.5	< 0.001	177
Cervix	20	−4.43 ± 0.68^Rb^.	< 0.001	71.9	< 0.001	1,107
Liver	6	−4.03 ± 1.37^Rb^.	0.003	81.8	< 0.001	59
Lung	12	−4.14 ± 0.71^Rb^.	< 0.001	62.9	0.002	382
Nerve	18	−2.91 ± 0.69^Rb^.	< 0.001	79.4	< 0.001	474
Prostate	4	−6.89 ± 1.1^Rb^.	< 0.001	-	0.498	58
Skin	42	−3.13 ± 0.24^Rb^.	< 0.001	32.6	0.024	4,251

The FsN value is considered robust (Rb.) if FsN ≥ 5k + 10, otherwise not robust (NRb.).

A-549, human lung cancer cell line; C6-Neural, murine glioma cancer cell line; CDOT, carbon dot of *C. limetta*; d_++_, Hedges’ mean difference; DU-145, human prostate cancer cell line; FsN, fail-safe N; HeLa, a human cervical cancer cell line; HepG2, human hepatoma cell line; I^2^heterogeneity derived from Cochran’s value; MCF-7, Michigan Cancer Foundation-7 (a human breast cancer cell line); MDA-MB-468, human breast cancer cell line; Melanoma, a type of skin cancer that originates from melanocytes; N, number of studies; NP, nanoparticle; NPC, nanocomposite; NPE, nanoemulsion; SE, standard error; UCC, untreated cancer cell.

### Dosages of the antioxidant and anticancer activities of CMNs

**Fig. 3:**
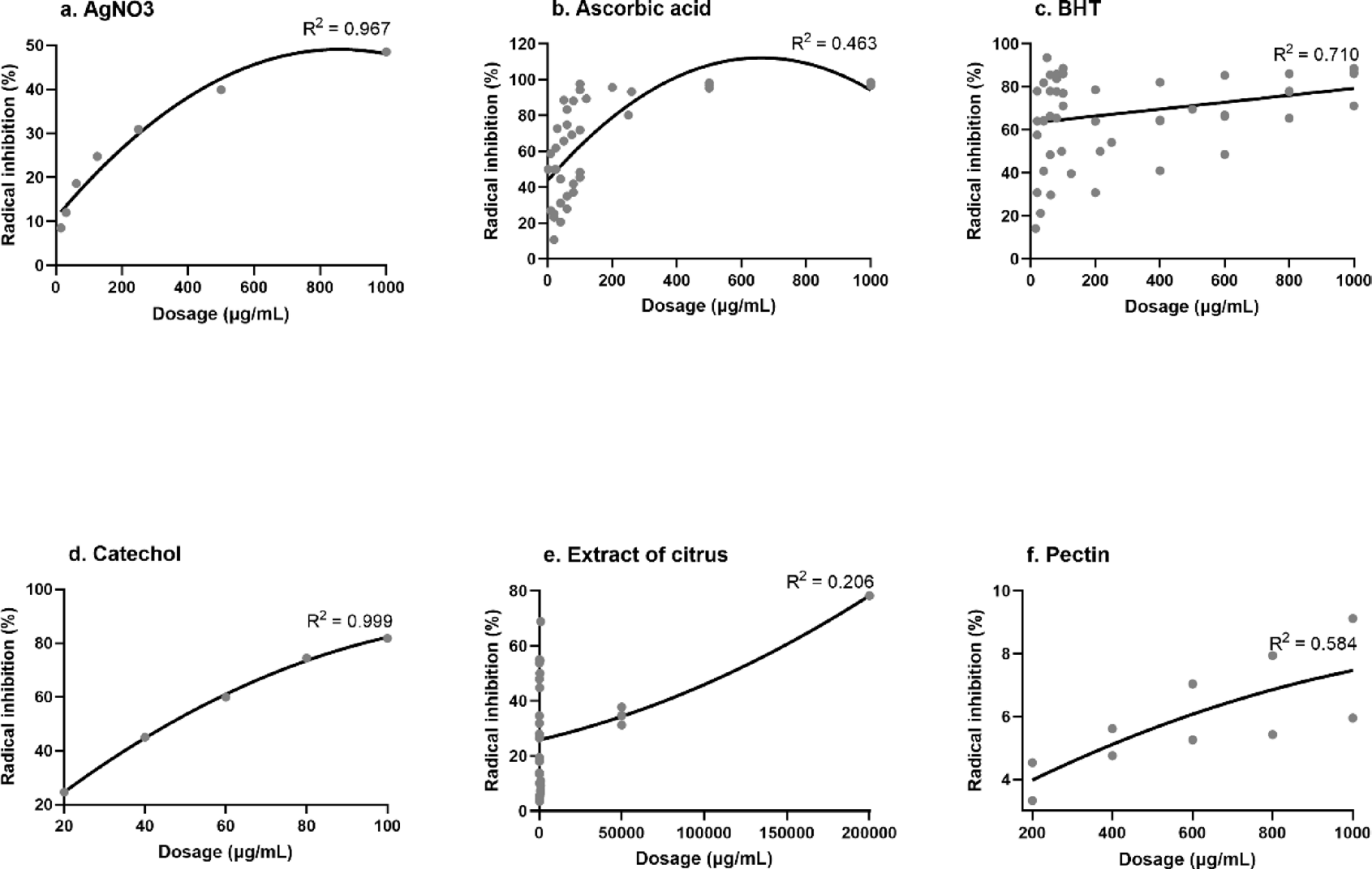
Effects of radical inhibition (%) from various control groups on antioxidant activity.

AsA and BHT inhibited free radicals by nearly 100% at concentrations below 200 µg/mL (Fig. 3). However, the data for AsA and was less consistent than those for BHT, with R^2^ values less than 0.75. Previous studies have confirmed that BHT had a stronger antioxidant activity than both AsA did^52^. Further, the IC₅₀ values (µM) in the DPPH assay were 16.1 for BHT, 23.8 for Trolox, and 39.4 for AsA^52^. Theoretical calculations also ranked the bond dissociation energy (BDE) in ascending order as BHT < Trolox < AsA^52^. BDE reflects an antioxidant’s ability to donate hydrogen atoms to neutralise free radicals^52,53^. A lower BDE corresponds to greater antioxidant potential^54,55^.

**Fig. 4:**
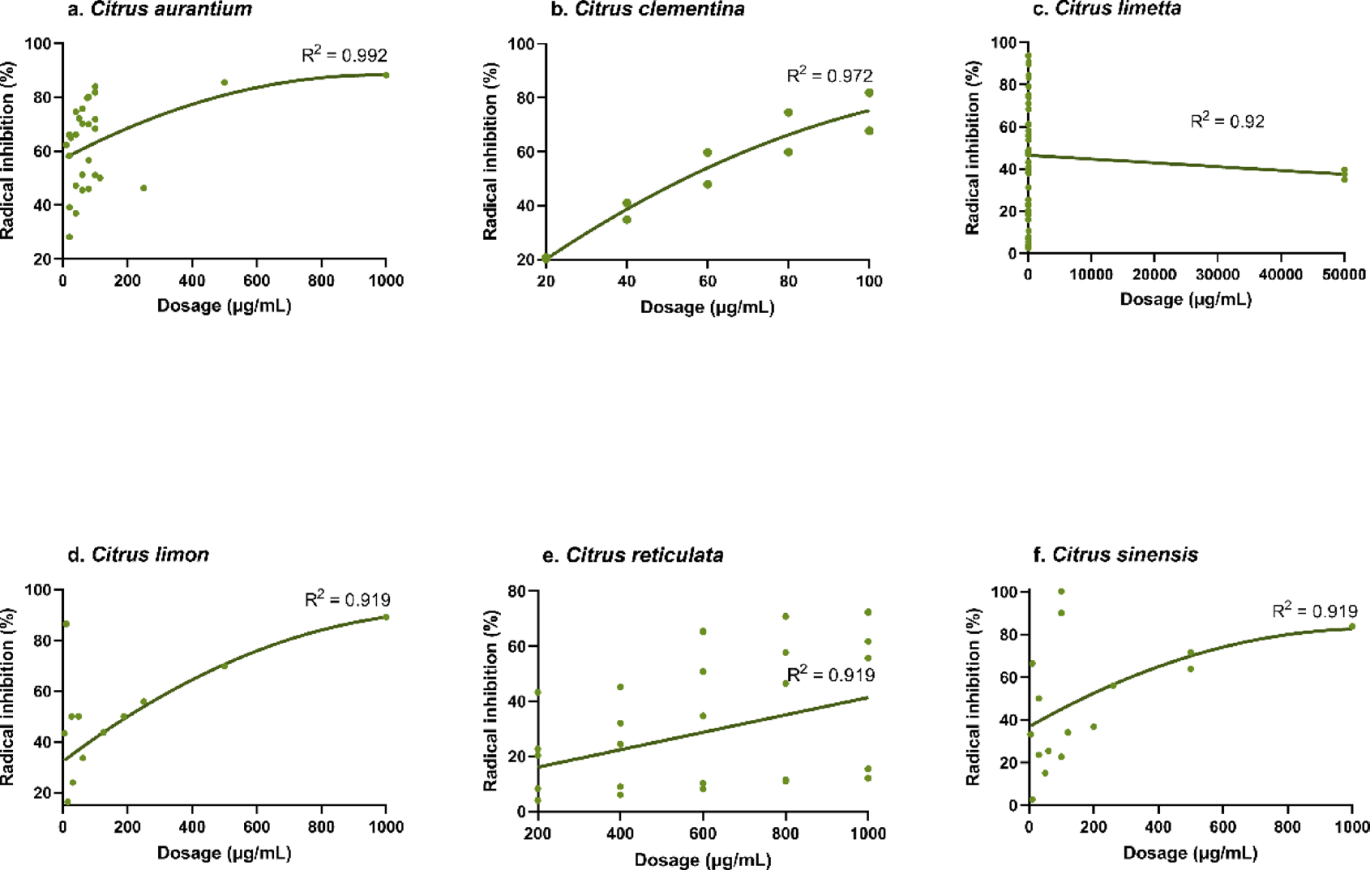
Radical inhibition (%) by different types of citrus-mediated nanoformulations.

Regression analysis indicated that CMNs generally presented a lower antioxidant capacity than the positive controls did (Fig. 3), with most effective antiradical doses exceeding 1,000 µg/mL (Fig. 4). However, CMNs derived from *C. clementina* and nanoparticles such as Au-NPs, CdO-NPs, and NiO-NPs showed relatively high radical inhibition at much lower doses (< 100 µg/mL), resulting in inhibition rates between 80% and 100%. In green synthesis, phytochemicals from *C*. *clementina* facilitated the formation of Ag-NPs with an average size of 17.5 nm. Moreover, the Au-NPs, CdO-NPs, and NiO-NPs were synthesised via phytochemicals extracted from *C*. *limetta*, *C*. *limetta*, and *C*. *aurantium*, respectively (Table 2).

**Fig. 5:**
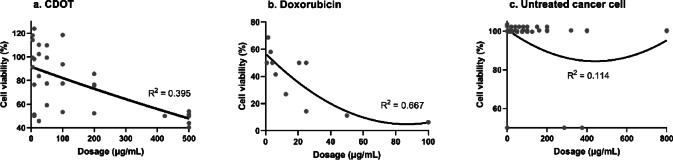
Cancer cell inhibition (%) by different control groups in terms of anticancer activity.

**Fig. 6:**
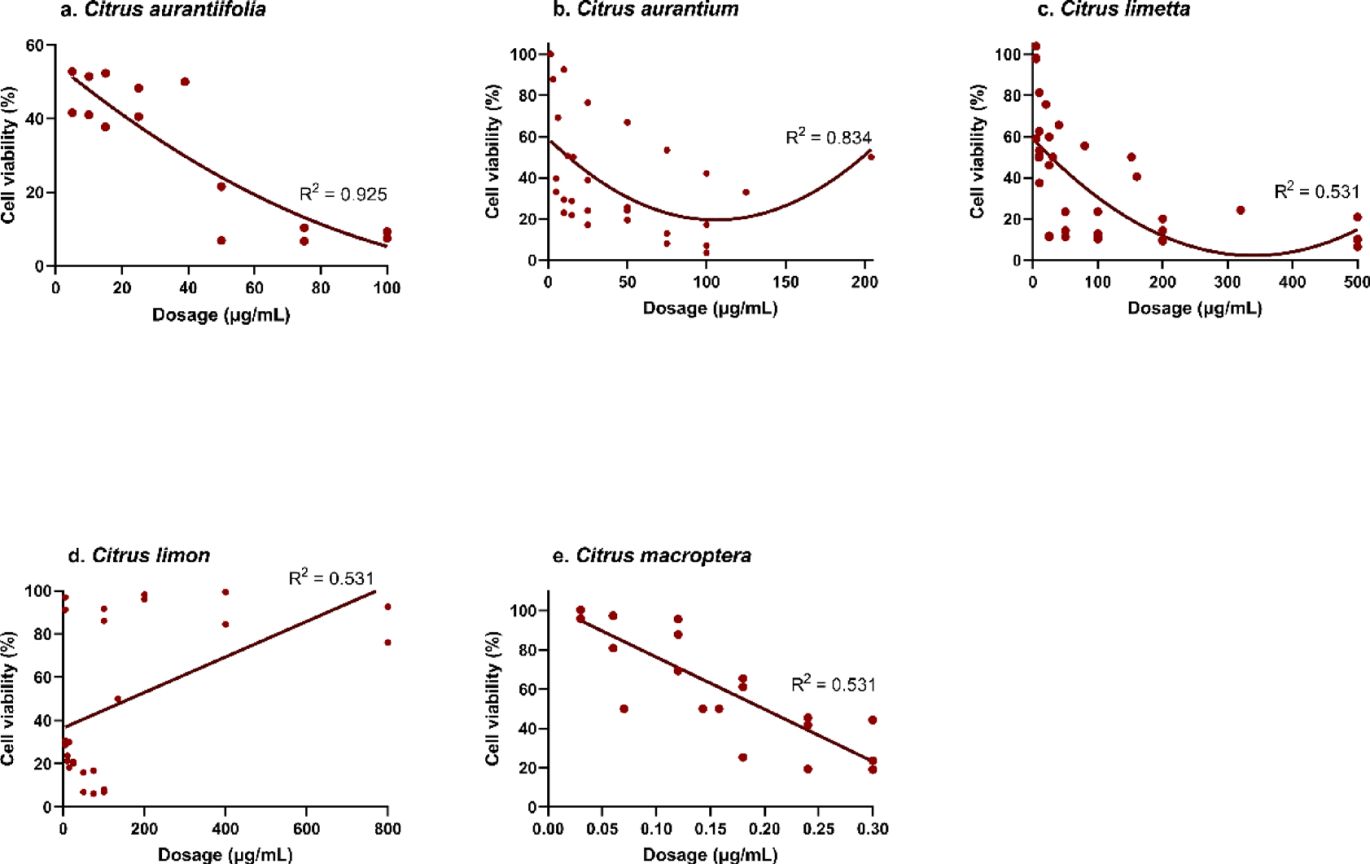
Cancer cell inhibition (%) by various citrus fruits.

Figure 5 shows the percentage inhibition of cancer cells by various control groups in relation to anticancer activity. The inhibitory effects of CMNs on cancer cells have been consistently demonstrated across multiple citrus species, including *C. aurantiifolia*, *C*. *clementina*, *C*. *limetta*, *C*. *limon*, *C*. *macroptera*, and *C*. *sinensis* (Fig. 6). These CMNs exhibit large effect sizes (d_++_ > 0.8), whether derived from juice or peel extracts. Compared to others, cerium oxide nanoparticles (CeO₂-NPs) most significantly reduce cancer cell viability. Specifically, CeO₂-NPs synthesised from *C*. *aurantiifolia* and averaging 22.5 nm in size, reduced the viability of HeLa (cervical cancer) cells by 7.5%, 23.5%, 33.1%, 46.6%, 57.8%, and 67.1% at concentrations of 10, 25, 50, 75, 100, and 125 µg/mL, respectively^34^. The reduction in cancer cell populations induced by CMNs surpasses that induced by carbon dots synthesised from *C*. *limetta* (CDOT), although doxorubicin continues to demonstrate superior efficacy^13,40,42,44,46^. Notably, the most pronounced anticancer response was observed in CMNs derived from *C*. *sinensis* peel extract, which achieved an 82.4% reduction in DU-145 (prostate cancer) cell viability^45^.

### Meta-network analysis of the effects of CMNs on variables related to antioxidant and anticancer activity

**Fig. 7:**
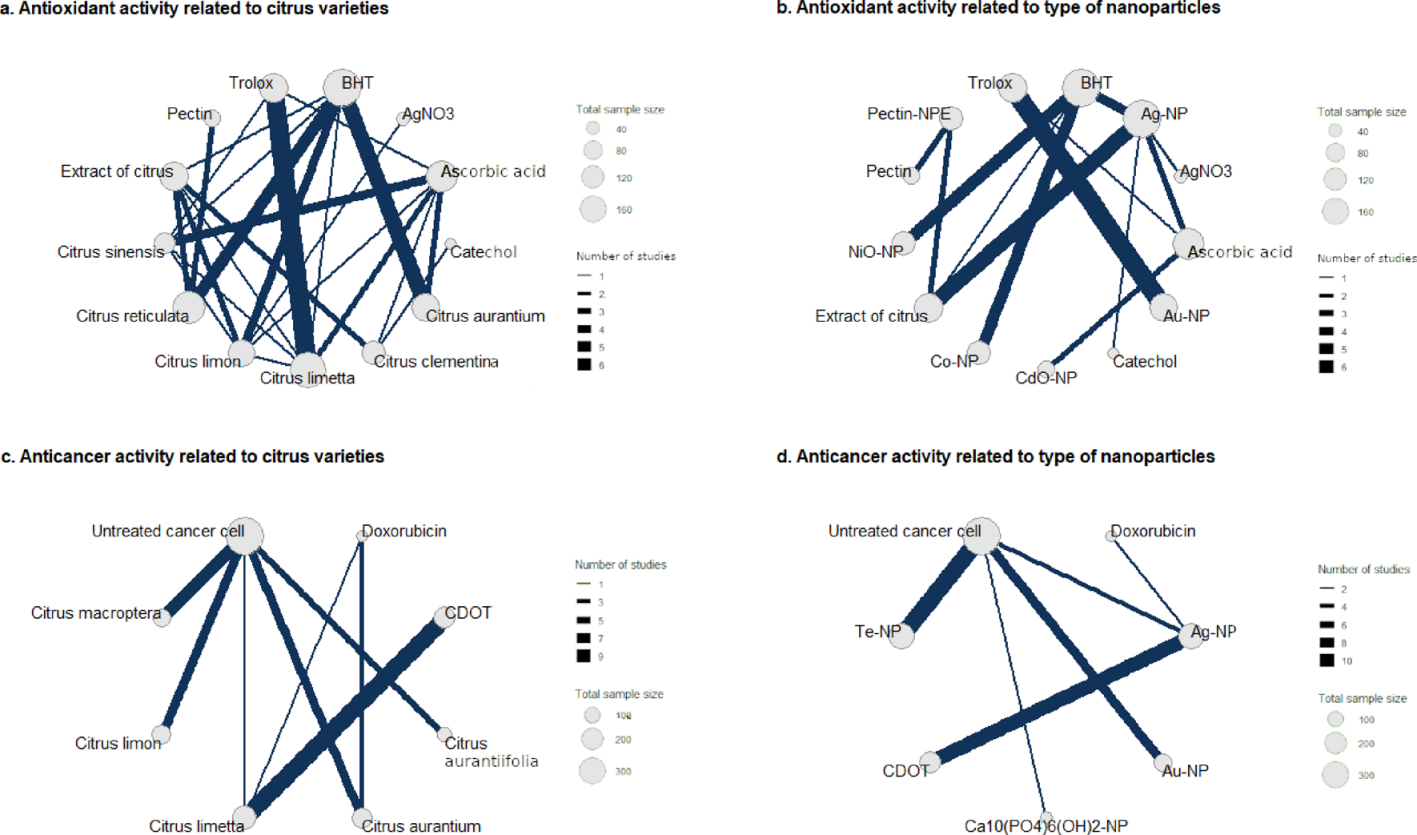
Meta-network analysis of the antioxidant and anticancer activities of citrus-mediated nanoformulations.

**Fig. 8:**
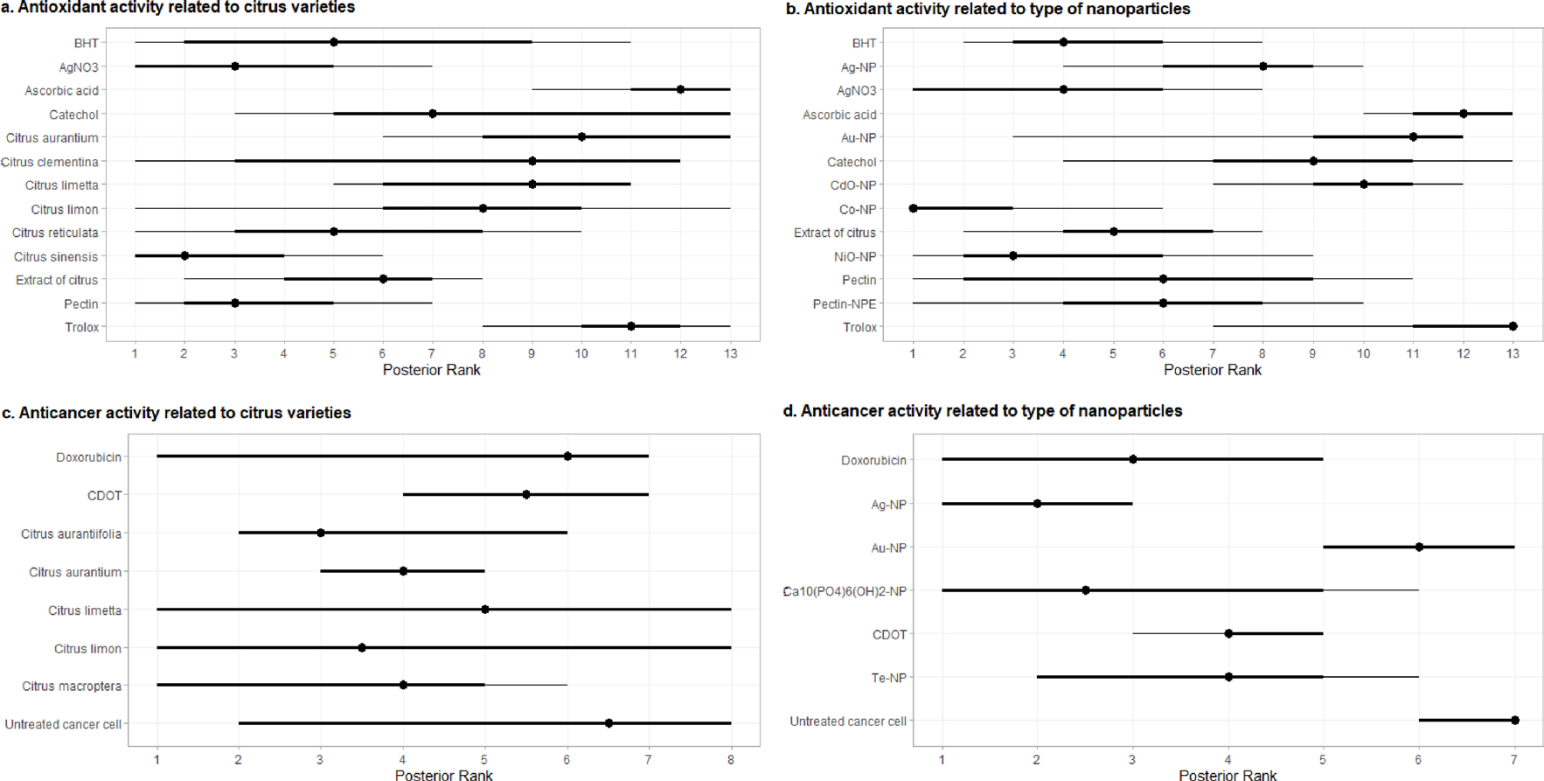
Meta-network analysis ranking of variables related to citrus-mediated nanoformulations in terms of antioxidant and anticancer activities.

The antioxidant activity extensively studied in the experiment involves comparisons such as Trolox (used as a positive control) versus CMNs derived from *C*. *limetta* and BHT versus CMN extracts from *C*. *aurantium*, *C*. *reticulata*, and *C*. *limon*. Additionally, minor discussions touch upon comparisons of AsA versus *C*. *sinensis* (Fig. 7a). With respect to nanoparticle types, comparisons include Trolox versus Au-NPs and the relationship between BHT and several CMN types, such as NiO-NPs, Co-NPs, and Ag-NPs (Fig. 7b). According to the rankings obtained from Fig. 8a, among the species categories, *C*. *sinensis* ranks highest compared with the other citrus varieties and positive controls (AsA, BHT, catechol, and Trolox). Following *C*. *sinensis* are *C*. *reticulata*, *C*. *limon*, *C*. *limetta*, *C*. *clementina*, and *C*. *aurantium*. The sequences of nanoparticle types were Co-NPs, NiO-NPs, pectin-NPEs, Ag-NPs, CdO-NPs, and Au-NPs (Fig. 8b).

Concerning the anticancer activities shown in Fig. 7c and d, the most frequent studies discuss the use of CDOT versus *C*. *limetta* as anticancer agents. Additionally, several studies have also examined UCC versus various citrus variants, such as *C*. *macroptera*, *C*. *limon*, *C*. *aurantium*, and *C*. *aurantiifolia*. Moreover, the most frequently studied nanoparticle types involve CDOTs versus Ag-NPs and UCCs versus various CMN variants, including Te-NPs, Au-NPs, and Ag-NPs. The anticancer effectiveness of CMNs based on species and nanoparticle type is discussed in Fig. 8c and d. Specifically, *C*. *aurantiifolia* showed the greatest effectiveness, followed by *C*. *limon*, *C*. *macroptera*, *C*. *aurantium*, and *C*. *limetta*. For the various nanoparticle types, the ranking order was Ag-NPs, Ca_10_(PO_4_)_6_(OH)_2_-NPs, Te-NPs, and Au-NPs.

## Discussion

### Antioxidant activity of CMNs

A lower IC_50_ value reflects greater potency in scavenging DPPH radicals, indicating greater antioxidant activity^56^. The meta-analysis results revealed a significant difference in the IC_50_ values between CMNs and the control group. The higher IC_50_ observed in the CMN treatment group suggested that the control had greater antioxidant activity. However, several studies have reported that CMNs exhibit lower IC_50_ values than citrus extracts do, indicating superior radical-scavenging capacity^57,58^. In contrast, other findings suggest that CMNs have higher IC_50_ values than citrus extracts do^59^. The antioxidant efficacy of CMNs largely depends on the redox potential of the capping agents, such as quercetin and gallic acid, present on the surface of Ag-Se nanoparticles^60,61^. These compounds function as potent reducing agents, hydrogen ion donors, and singlet oxygen^1^O_2_) scavengers, thereby enhancing the antioxidant performance of CMNs^62^. Additionally, phytochemicals in Citrus sinensis contribute to the neutralisation of reactive nitrogen species (RNS) and reactive oxygen species (ROS)^63^. In advanced applications, juice from *C*. *limon* has been used to form nanovesicles that protect cells from oxidative stress. This protection occurs through the activation of the aryl hydrocarbon receptor (AhR) and nuclear factor erythroid 2-related factor 2 (Nrf2) signalling pathways^64^.

The antioxidant results, based on the IC_50_ values, revealed a significant effect, particularly in CMNs derived from citrus peel extracts. Subgroup analysis confirmed that only peel-derived CMNs demonstrated statistically significant differences. This notable potential may stem from the high flavonoid content in the peel^65^. Flavonoids are predominantly located in the flavedo and albedo layers of citrus peels, whereas carboxylic acids are mainly found in the fruit segments^66^. Citrus peels are rich in bioactive compounds, making them a sustainable and renewable source of phenolics^65^. Compared with the pulp and seeds, the peel contains higher concentrations of phenolic compounds and exhibits greater antioxidant activity^67^. In addition to the type of citrus species and the plant part used, the geographical origin of the citrus is crucial. Different citrus species, depending on their location, present varying levels of antioxidants, flavonoids, and phenols^68,69^. Extraction techniques influenced the concentration of antioxidant compounds, as indicated by a meta-analysis that reported a weak correlation for citrus extracts (R² = 0.206; Fig. 3e). In contrast, standard controls commonly used in antioxidant evaluations showed stronger regression values, including AsA, BHT, and catechol (Fig. 3b–d). Incorporating citrus extracts into CMN-based compounds improved their bioavailability as radical-scavenging agents, particularly those derived from *C. aurantium*, *C. limon*, *C. clementina*, *C. reticulata*, and *C. sinensis*, all of which showed R² > 0.75. An exception occurred in *C. limetta*, which exhibited a decreasing trend with R² = 0.92 (Fig. 4). Previous findings supported this observation, where green synthesis modification of C. aurantium peel extract produced a DPPH IC₅₀ value of 19 µg/mL, which surpassed BHT^37^. Although BHT showed much stronger oxidative power than AsA, BHA, and Trolox^52^, the findings remained promising. The abundant availability of citrus peel waste in nature indicated antioxidant potential comparable to BHT, as previously reported.

The radical inhibition of the CMN was slightly greater than that of the control, with the strongest to weakest order being Ag chitosan-NP > Whey-NPE > Pectin-NPE > Hydrogel-NPC > CeO2-NP > TiO2-NP > Ag-NP > Fe3O4-NP > Au-NP > NiO-NP > Co-N > CdO-NP > Au@Fe2O3-NPC > V-NP. The ratios of Fe3O4-NPs to V-NPs were lower than those of the control. It would be helpful to explain the pros and cons related to nanoparticles based on the previous statement, such as whether Ag chitosan-NPs truly have a greater antioxidant capacity. This discovery confirms that nanoparticles derived from citrus phytochemicals can be used as antioxidants in various applications. However, to fully understand the nature, physicochemical properties, and mechanisms of action of nano-antioxidant composites, it is crucial to study their catalytic and biological activities^62^. Phytochemical substances are crucial reducing agents and stabilizers in nanoparticle synthesis^22,59,70^. Each substance has a distinct function. Alkaloids serve as reducing agents, flavonoids and terpenoids act as stabilizers and capping agents, and proteins and carbohydrates function as both stabilizers and reducing agents in the transformation of metallic salts into metallic nanoparticles^71^. Finally, nanoparticles with biologically active phytochemical components exhibit bacteriostatic properties due to various functional groups, such as OH, NH_2_, COOH, and NO_2_^72–74^.

The therapeutic efficacy of CMNs strongly depends on the physicochemical characteristics of their constituent metals. Studies have shown that nanoparticles synthesised from *Clerodendrum inerme* (CI) leaf extracts using gold (Au) and silver (Ag) exhibit different levels of antioxidant activity^75^. Specifically, CI-Ag-NPs demonstrate a higher DPPH scavenging rate than CI-Au-NPs do, with values of 78.8% and 75.9%, respectively^75^. Further evidence suggests that Ag-NPs prepared with 2.5 mM gallic acid possess a greater absolute zeta potential than do Au-NPs, with values of 40 mV and 38 mV, respectively^76^. A higher absolute zeta potential enhances the stability of nano-antioxidant systems^77–79^. Researchers have successfully incorporated natural antioxidants into nanospheres by forming complex covalent bonds between metal ions and phytochemicals, thereby improving their antioxidant functionality^80^. This bonding also contributes to nanoparticle stability^59,81^. Moreover, phytochemical extracts can neutralise the intrinsic oxidative properties of nanoparticles, enabling their antioxidant activity^82^. Numerous studies support these observations. For example, one investigation reported that CMNs exhibit substantial antioxidant capacity, with ABTS and DPPH assay results comparable to those of the standard antioxidant Trolox^58^. In contrast, another study revealed no significant differences in antioxidant activity between various lemon essential oils and their corresponding nanoemulsions, as measured by both ABTS and DPPH assays^5^. Furthermore, compared with those produced via conventional synthesis, zinc oxide nanoparticles synthesised via green methods with *C*. *limetta* peel extract demonstrated superior antioxidant capacity^35^. Another finding concerns the green synthesis of silver nanoparticles using extracts from *Punica granatum* and *Plectranthus rugosus*, which demonstrated free radical scavenging activity against 2,2-diphenyl-1-picrylhydrazyl at 70% and 68%, respectively^83^. In addition, extracts from *Azadirachta indica* have also been reported to possess antidiabetic properties^84^.

### Anticancer activity of CMNs

Differences in cell viability responses are likely due to the ability of CMNs to reduce cancer cell viability. In a subgroup meta-analysis, several citrus species and specific plant parts, such as juice, had significant positive effects on cancer inhibition, with *C*. *macroptera* and nanoparticles of certain sizes demonstrating strong results. The synthesis of nanoparticles throughout the study revealed that the green synthesis used to obtain CMNs has biological properties as an anticancer agent^40^. Previous findings indicate that nanoparticles from *C*. *macroptera* incorporated with gold (Au) at a dose of 300 ng/mL significantly reduce the populations of A-549 (lung cancer), MDA-MB-468 (breast cancer), and HepG2 (liver cancer) cells^16^. The size of *C*. *macroptera* nanoparticles is estimated to be approximately 31 nm via DLS tools^16^. Moreover, *C*. *macroptera* has an IC_50_ value of 70.2 ng/mL against HepG2 cells, while the values for A549 and MDA-MB-468 cells are 143 ng/mL and 157.9 ng/mL, respectively^16^.

Other findings also indicate that CMNs from *C*. *aurantium*, *C*. *aurantiifolia*, and *C*. *limon* combined with Te-NPs (101, 150, and 150 nm, respectively) have IC_50_ values of 204, 39, and 135 µg/mL against melanoma^15^. A size range of 101–500 nm was confirmed to be the most effective anticancer agent. Additionally, the incorporation of *C*. *aurantium* extract with V-NPs 80 nm in size has an IC_50_ of 15.9 µg/mL against MCF-7 (breast cancer) cells^13^. Furthermore, Cd-NPs from *C*. *limetta* (peel extract) and Ag-NPs from *C*. *clementina* (peel extract) had IC_50_ values of 152.2 (against A-549, lung cancer) and 60 µg/mL (C6-Neural, nerve cancer), respectively. Although the meta-analysis model confirmed that only the inhibition of A-549 cells and melanoma by CMNs was robust, data supporting other cancer cell models remain scarce.

**Fig. 9:**
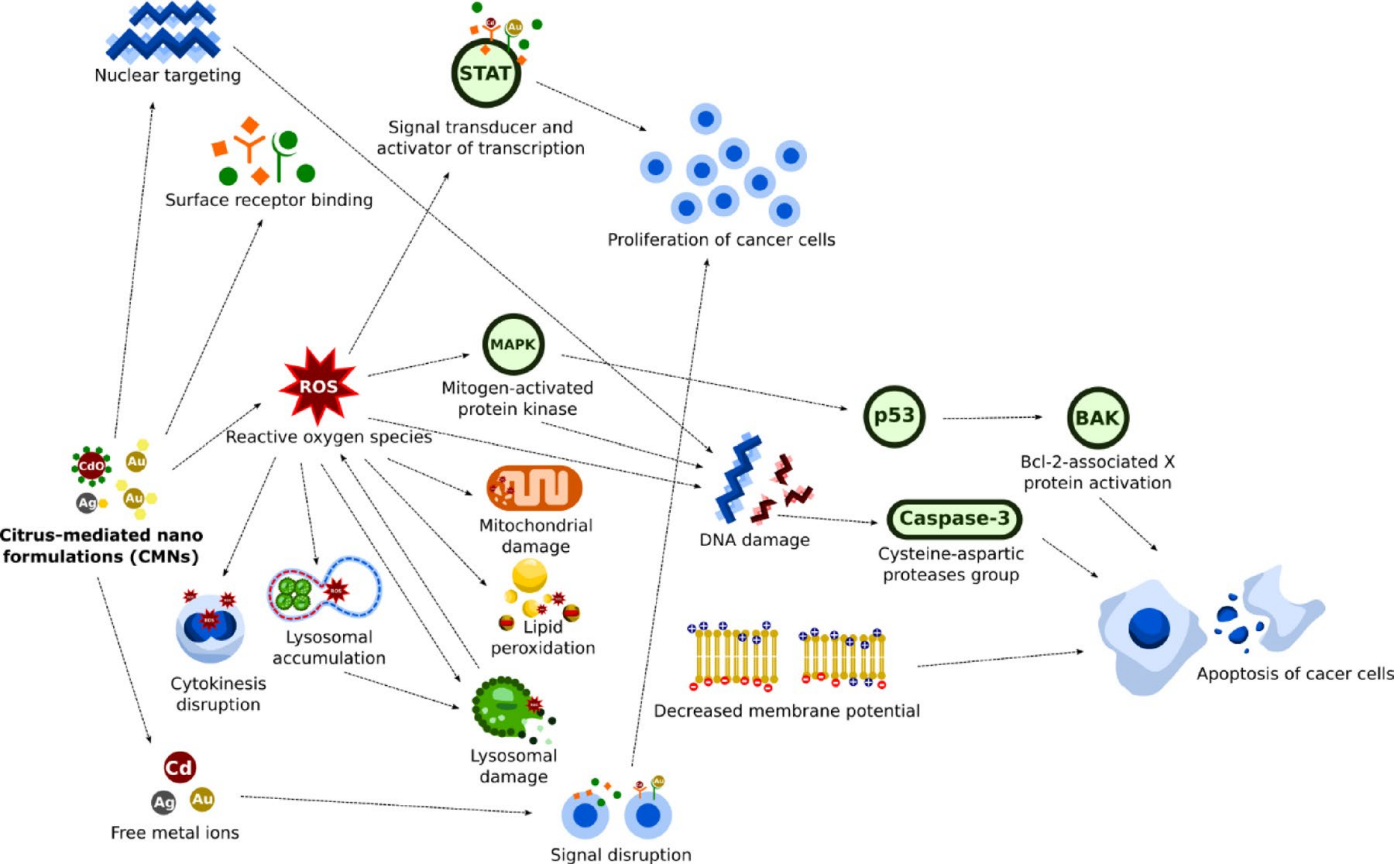
Molecular pathways of cytotoxicity and immunomodulation induced by citrus-mediated metal nanoparticles in cancer therapy^99–102^.

The cytotoxicity to cancer cells caused by CMNs is attributed to two primary factors: particle aggregation and ROS formation. Particle aggregation triggers lipid beta-oxidation in the cell membrane, leading to increased cell viability (Fig. 9)^16^. ROS can induce apoptosis, which has been observed in DU-145 cells treated with Ag-NPs (*C*. *sinensis*)^45^. Additionally, the antioxidant properties of V-NP from *C*. *aurantium* are believed to involve alkaloids, ascorbic acid, flavonoids, saponins, and tannins that are correlated with DPPH. V-NP also scavenges free radicals, which can trigger mutagenesis and proliferation of cancer cells^13^. The results of previous experiments clearly demonstrated that nanoparticles derived from *Olea paniculata* and *Bauhinia variegata* Linn extracts exhibit increased antioxidant activity, which strongly correlates with the concentration of the extracts^85,86^. Other possible mechanisms resulting from the use of nanoparticles as cancer cell inhibitors include DNA damage, mitochondrial disruption, disturbance of cellular signalling pathways, lysosomal dysfunction, increased free metal ions, and autophagy (Fig. 9).

Au-NPs targeted at the cancer cell nucleus can cause significant DNA damage, leading to apoptosis (Fig. 9). Au-NPs disrupt cytokinesis, resulting in binucleated cells and increased double-strand DNA damage^87^. Similarly, iron oxide nanoparticles can also induce apoptosis in cancer cells through oxidative stress, which includes increased ROS and lipid peroxidation, as well as reduced antioxidant activity. Oxidative stress causes DNA damage, which can be detected through comet assays and chromatin condensation. The activation of caspase-3 indicates the apoptotic pathway, resulting in programmed cell death due to DNA damage. Thus, iron oxide nanoparticles induce apoptosis in cancer cells through oxidative stress mechanisms that cause DNA damage^88^. Furthermore, ROS from metal ion nanoparticles can also induce mitochondrial dysfunction in cancer cells. Such damage includes decreased mitochondrial membrane potential, which is essential for ATP synthesis and cellular homeostasis. This process induces the expression of proapoptotic genes such as p53 and bcl-2-associated X protein activation (BAX). Mitochondrial damage also reduces the number of healthy mitochondria, alters mitochondrial morphology, and causes broader dysfunction in cancer cells^89,90^. Additionally, when nanoparticles accumulate in lysosomes, their pH can be altered, leading to lysosomal damage and increased ROS production^91^. This generates oxidative stress that can damage cellular components and trigger apoptosis^91,92^. Moreover, the increase in free metal ions released from nanoparticles can exacerbate oxidative stress and influence signalling pathways that regulate cancer cell growth^93^. Nanoparticles can also induce or disrupt autophagy, which is crucial for maintaining cellular homeostasis^94^. Therefore, while nanoparticles can provide significant therapeutic effects, they may also contribute to cancer therapy resistance, depending on their physical and chemical characteristics^94,95^.

Nanoparticles can affect cellular signalling pathways through various mechanisms (Fig. 9). First, they can bind to receptors on the cell surface, triggering cell proliferation or death. Second, they increase ROS production, which can activate signalling pathways such as the MAPK and STAT pathways, which play roles in cell growth, proliferation, and death^96,97^. Additionally, nanoparticle exposure can lead to cell death through apoptosis pathways, depending on the concentration and duration of exposure. Nanoparticles also affect chaperone proteins and the p53 gene, which are involved in DNA repair and cell cycle regulation. Finally, magnetic nanoparticles can activate signalling pathways by applying physical forces to cells. Overall, interactions between nanoparticles and cells can lead to significant changes in cellular function and health, necessitating further research to understand their long-term effects^96,98^.

Potential CMNs as anticancer agents also raise concerns regarding their cytotoxicity toward healthy cells. Reports suggest that they can induce oxidative stress by increasing ROS production and damaging DNA, lipids, and proteins^103,104^. Furthermore, nanoparticles may disrupt cell membrane integrity, potentially leading to cell death^63,105,106^. Among CMNs, Au-NPs, Te-NPs, and larger particles strongly inhibit cancer, while certain sizes of CMNs have significant effects. Citrus extracts are also effective as coatings, enhancing nanoparticle efficacy. Positive controls such as doxorubicin show notable results, whereas some negative controls show the opposite results, underscoring the importance of composition. These findings support the therapeutic potential of CMNs in cancer treatment, although further research is necessary to refine their properties and applications.

## Conclusion

This study reveals that various types of citrus-mediated nanoformulations (CMNs) display distinct antioxidant and anticancer activities. Among them, *C. reticulata* and *C. sinensis* exhibit the highest free radical scavenging potential. In contrast, CMNs synthesised from *C. aurantiifolia* and *C. macroptera* show superior anticancer effects, particularly against specific cancer cell lines. Particle size also plays a crucial role, with CMNs ranging from 101 to 500 nm being more effective at neutralising free radicals, while those ranging from 51 to 100 nm better inhibit cancer cell proliferation. In terms of nanoparticle type, gold-iron oxide nanocomposites (Au@Fe₂O₃-NPC), cadmium oxide nanoparticles (CdO-NPs), hydrogel-based nanocomposites (hydrogel-NPC), and vanadium nanoparticles (V-NPs) demonstrate strong antioxidant capacity. Moreover, silver nanoparticles (Ag-NPs), cerium dioxide nanoparticles (CeO₂-NPs), and V-NPs have notable cytotoxic effects against cancer cells. Nevertheless, the bioactivity of these CMNs remains lower than that of standard controls such as butylated hydroxytoluene (BHT) and doxorubicin. Despite these limitations, CMNs demonstrate considerable promise in suppressing the proliferation of various cancer cell lines, including human lung carcinoma (A549), rat glioma (C6), human prostate carcinoma (DU-145), human cervical cancer (HeLa), human hepatocellular carcinoma (HepG2), human triple-negative breast cancer (MDA-MB-468), and melanoma cells.

This meta-analysis has several limitations. The primary constraint lies in the efficacy effects on living organisms, which still require clinical validation. This limitation restricts the generalisability of the findings. In addition, despite the focus on a single species and the use of citrus fruit extracts, substantial heterogeneity remains across the included studies. A broader exploration of bioactive compound evaluations is necessary to identify consistent metabolomic patterns across different citrus extracts, which may vary depending on extraction techniques, sample types, and environmental conditions.

Therefore, further research is essential to address the existing gaps concerning the toxicity of CMNs, particularly to support their practical application in medical fields. Fundamental molecular assays and detailed investigations into the mechanisms of action of CMNs are also needed to better understand their efficacy and potential side effects. Furthermore, the standardisation of CMN processing protocols is crucial, especially considering their prospective use in pharmaceuticals and even functional foods, to ensure both effectiveness and product safety.

## Data Availability

The datasets used and/or analysed during the current study available from the corresponding author on reasonable request.
